# Multilingual voice-enabled informatics tools: Catalyst for equitable AI in HIV and HIV-comorbidity healthcare management

**DOI:** 10.1371/journal.pone.0332573

**Published:** 2025-10-21

**Authors:** Olugbenga Oluseun Oluwagbemi, Emebo Onyeka, Abdulwahab Jatto, Folakemi E. Oluwagbemi, Serengul Smith, Olufemi Oyelami, Segun Fatumo

**Affiliations:** 1 Department of Computer Science, Faculty of Science and Technology, Middlesex University, The Burroughs, Hendon, London, United Kingdom; 2 Department of Computer Science, Virginia Tech, Blacksburg, Virginia, United States of America; 3 Department of Computer Science, Federal University Lokoja, Lokoja, Nigeria; 4 Faculty of Health, Social Care & Education, Middlesex University, The Burroughs, Hendon, London, United Kingdom; 5 Computer Science Programme, Bowen University, Iwo, Nigeria; 6 Department of Medicine, Precision Healthcare University Research Institute, Queen Mary University of London Empire House, London, United Kingdom; Kampala International University - Western Campus, UGANDA

## Abstract

Human Immunodeficiency Virus (henceforth HIV) is a global health problem, presently with no known cure. Africa has one of the highest incidences of HIV. Nigeria, within the West African (WA) region, is one of the largest economies on the continent. However, the country continues to struggle with HIV, with approximately 2 million individuals currently infected and experiencing ongoing transmissions. Management of the disease has been difficult due to communication barriers between English-speaking medical practitioners and indigenous patients in rural and suburban regions of the country and bordering countries. In this paper, we used fuzzy logic and voice-enabled technology to create WAHMIDS (West African HIV and HIV-comorbidity Multilingual Indigenous Diagnostic Software) and WAHMIMA (West African HIV Multilingual Informatics Mobile Application), which are health apps designed to help diagnose HIV and manage related health issues in both rural and urban areas for people who speak different indigenous languages in West Africa. Additionally, illustrations of the application of this tool to HIV diagnosis, using existing HIV data, are demonstrated. We expect that these tools will assist English-speaking medical workers and inhabitants of West African communities in their efforts to control HIV transmissions. These informatics tools have the potential to help prescribe medications for HIV and HIV-comorbidity patients. We anticipate that these informatics tools will help address healthcare disparities and promote diversity, equality, and inclusion by reducing the gaps in healthcare delivery between different regions and facilitating the collection of diverse patient data, which is essential for developing and planning more inclusive and accurate healthcare strategies in the West African sub-region.

## 1. Introduction

A primary obstacle in addressing diseases in the West African subregion is the linguistic barrier resulting from the multitude of indigenous languages spoken throughout the region. These languages are commonly used in both countryside and city areas, and the differences between them have led to delays in HIV diagnoses and limited help for people with HIV and related health issues in rural and semi-urban areas [1–7]. West Africa hosts numerous indigenous languages, notably Yoruba, Hausa, and Igbo, which are prevalent in multiple countries within the region. The Yoruba language is primarily spoken in Southwestern Nigeria, with substantial numbers in other West African nations, including Ghana, the Republic of Benin, Togo, and Sierra Leone [8–15]. Similarly, numerous rural regions across various West African countries extensively utilize Igbo and Hausa as local languages [16–19].

Another barrier to the fight against HIV is the emigration of medical professionals out of the West African region to seek better remunerations and opportunities in advanced countries [16–19]. In the fight against HIV, English-speaking medical doctors whose places of duty are in rural communities of West African countries frequently have difficulty communicating with local patients during HIV diagnosis and HIV-comorbidity treatments. Occasionally there is no communication at all [20]. Generally, a lack of proper communication channels is considered an impediment to the effective treatment and management of HIV and HIV-comorbidity in resource-poor communities and resource-limited settings [21]. Many rural areas in West African countries and many communities in Nigeria often use Yoruba, Igbo, and Hausa as their major indigenous languages of communication. The major languages of communication are also common at rural health centers [16–19]. These language limitations waste time and effort and at times lead to delayed treatments and diagnoses. These limitations significantly affect English-speaking medical personnel working in these regions, particularly when interpreters are unavailable due to limited resources. Communication barriers can result in time wastage, frustration, delayed treatments, and, in extreme cases, without HIV test kits, wrong diagnoses. The emigration of medical doctors and personnel has led to the problem of brain drain [22–26]. Brain drains have led to an insufficient number of university lecturers, medical experts, and medical doctors in rural areas to manage HIV care and HIV-comorbidity treatments [27–36]. Brain drains have led to a scarcity of university medical specialists, consultants, and medical personnel who can help manage cases of HIV patients. The third problem is the lack of sufficient HIV testing kits and services. This scenario is especially common in rural areas [37]. Knowledge of HIV-positive status among inhabitants of some West African countries is low [38,39]. An estimated 25.6 million people live with HIV in Sub-Saharan Africa, a representation of two-thirds of the global total [40]. Currently research indicates that 60% of people living with HIV (PLWHIV) are aware of their status. The remaining 40% are ignorant of their HIV status, perhaps due to their inability to access proper HIV testing services. Many West African rural and suburban dwellers lack access to HIV diagnostic and testing services. For instance, there is a high incidence of HIV in the Northern region of Nigeria, as well as in the Southeast region of the country [41–45]. West African countries like Ghana, Togo, the Republic of Benin, and Sierra Leone also experience some incidences of HIV and increased new HIV infections, particularly in rural and suburban areas where locals speak indigenous languages such as Yoruba, Hausa, and Igbo [46–60]. In these rural and suburban regions, there are insufficient HIV test kits, insufficient provision of antiretroviral therapy (ART) to the HIV population, expensive costs of HIV test kits, and low levels of literacy. These obstacles hinder the effective management of the disease [61–63].

All these problems have resulted in the loss of lives, shortage of manpower, delayed HIV diagnosis, delayed HIV-comorbidity treatments, and delayed management of HIV in poor communities and low-resource/resource-limited settings. The objective of this research is to develop an indigenous voice-based HIV diagnostic software called the West African HIV and HIV-comorbidity Multilingual Indigenous Diagnostic Software (WAHMIDS, hereafter) and WAHMIMA (West African HIV Multilingual Informatics Mobile Application) in English and prominent indigenous West African languages. These informatics tools aim to complement medical personnel to efficiently diagnose HIV and manage HIV-comorbidity patients in rural, suburban, and urban West African communities. It will also assist with HIV staging. The voice-enabled mobile application will also help the inhabitants of different rural and suburban regions of West Africa acquire the appropriate knowledge about HIV transmissions, symptoms, and prevention. The justification for developing these informatics tools stems from the existing scarcity of multilingual health informatics resources that meet the demands and needs of medical doctors who care for HIV and HIV-comorbidity patients in indigenous communities across the West African region. One of the major communication barriers is the indigenous languages spoken in these communities. This research is significant as it provides support and addresses the challenges medical personnel encounter when communicating with locals, which hampers the rate of HIV diagnosis and HIV-comorbidity management in the region. The novel informatics tools produced from this research will help address these challenges. Second, to achieve equitable representation in the design and development of multilingual applied health informatics tools, it is paramount to prioritize inclusiveness and promote the use of indigenous languages in developing applied health informatics software, especially for underrepresented communities. Third, the issue of healthcare disparities can be addressed, which will promote diversity, equity, and inclusion in the distribution of health resources and the adoption of health informatics tools. Finally, the implementation of these informatics tools will ultimately lead to the collection of diverse anonymous patient data, which will be essential for developing more robust, inclusive, and accurate healthcare strategies.

This study poses the following research questions: (i) How Can medical personnel working in local communities in West Africa be aided in overcoming language communication barriers and supported in achieving effective HIV and HIV-comorbidity diagnosis and management? (ii)) What computational methods can be explored and applied to complement the activities of English-speaking medical personnel in the effective diagnosis and management of HIV and HIV-comorbidity among indigenous residents of West African rural, suburban, and urban communities? iii) How can the adoption and utilization of health informatics tools be used to promote health management, diversity, equality, and inclusive care among the African HIV and HIV-comorbidity population?

We conducted a systematic literature review on articles spanning the year 1999–2024, on Google Scholar database, with search criteria focusing on published research articles on HIV that produced HIV systems that focused on management of HIV cases (See S1 Fig in S2 Appendix). The following search terms were used: “Fuzzy set AND fuzzy logic AND HIV diagnosis AND HIV symptoms AND diagnosis AND multilingual AND indigenous AND diagnostic systems”. We were able to screen and exclude research papers that did not fit into our search criteria. We identified 8 research manuscripts that matched our search criteria as summarized in S1 Table.

Ou literature search focused on research articles with emphasis on Fuzzy set, fuzzy logic HIV diagnosis, HIV related software. Some researchers developed a fuzzy logic multilingual indigenous diagnostic system (MAVSCOT) was developed to address the limitations associated with the management of HIV in rural South Africa [64]. MAVSCOT’s limitation is that it can only be used in the South African subregion where indigenous languages are spoken or understood. Another limitation of MAVSCOT is that it did not fully address the issues of HIV-comorbidities. The application of MAVSCOT failed to effectively manage patients with Hepatitis-HIV and other HIV comorbidities. MAVSCOT lacked a mobile application version. The indigenous languages in MAVSCOT cannot address the West African language barriers and challenges faced by HIV medical personnel within the West African sub-region. Thus, the need to develop region-specific multilingual indigenous HIV and HIV-comorbidity informatics diagnostics software for the West African subregion and the corresponding voice-enabled mobile application. Previous research also indicated that fuzzy cluster means algorithms have been used to develop an intelligent diagnostic system for HIV/AIDs [65]. Pazzani and colleagues [66] applied rule-based expert systems to managing HIV-infected patients. The system they developed encodes information from drug-resistant mutation literature. Their system did not comprehensively establish the link between genomic mutations that generate drug resistance and the corresponding surrogate outcomes. The research conducted by Ojeme and Maureen [67] helped develop an HIV diagnosis system that incorporated elements of Fuzzy logic, fuzzy sets, and neural networks. Their work created a model that helps identify the risk levels of patients living with HIV. T The results produced from their research revealed that the tool is user-friendly and generates diagnostic results. However, their informatics tool has fewer HIV symptoms. Another study developed an interactive and web-based expert system for HIV patient care. The system adopts a methodology that integrates HTML and CGI-script for implementation [68]. There is no mention of being able to make comprehensive predictions. Joglekar and colleagues [69] implemented a predictive algorithm to perform analysis on HIV data sets within the medical sector. Data containing 75 patient records was obtained from a local hospital in Mumbai, India. Their system accepts data as follows: patients record data about HIV symptoms; patients’ basic data, such as age and gender, among others; and patients’ history and previous or present lifestyle. The predictive algorithm within their system can predict the possible percentage of HIV present in patients during prediction. One limitation of their system is the limited number of HIV symptoms that are integrated into it. Seidenberg and colleagues developed a mobile-based SMS system for diagnosing HIV infection in early infants in Zambia [70]. Their mobile-based system has the capability of disseminating blood test results to laboratories by text messaging, where HIV screening takes place. A web-based HIV/AIDS medical consulting system was developed by Ebrahimi and colleagues [71]. Their system has the capability of providing consulting services based on available relevant input data. An HIV Interactive telephone-based Voice Response self-monitoring system was developed by Tucker and colleagues [72]. Their system was useful in assessing daily adherence to HIV antiretroviral medication. One of the limitations of their system was that it was not a diagnostic system and could not validate the expiration of HIV antiretroviral medication.

Uncertainties are associated with the decision-making process within the medical field. Fuzzy logic and fuzzy sets have played significant roles in resolving such uncertainties. In the research conducted by Deepa and colleagues [73]. The knowledge of intuitionistic fuzzy sets was applied to medical diagnosis. However, Deepa and colleagues’ system lacks multilingual capabilities and does not specifically target HIV diagnosis. Another study by Janghorbani and Moradi [74] applied the knowledge of fuzzy networks to resolve uncertainties in health prognosis and diagnosis. Hooda and Kumari [75] improved medical diagnosis by utilizing the knowledge of fuzzy soft sets. Additionally, Dutta and Doley applied the knowledge of bipolar-valued fuzzy sets to resolve uncertainties and improve medical diagnoses [76]. Their findings align with the results obtained from human reasoning and analysis. Riaz and Tehrim [77] employed bipolar fuzzy soft mappings to enhance the diagnosis of medical patients living with bipolar disorder. Furthermore, Mehmet [78] successfully employed fuzzy sets to distinguish between normal and diseased cat hearts. Precup and colleagues [79] improved the finger dynamics performance of a myoelectric-based management system for a prosthetic hand by applying computer algorithms to generate fuzzy models, achieving notable success. Oluwagbemi and colleagues [80] developed a voice-enabled antimalarial drug informatics system based on the knowledge of fuzzy logic, which helps to reduce uncertainties in the predictive system, but the informatics also lacks multilingual capabilities. Similarly, Oluwagbemi and colleagues [81] utilized fuzzy logic and knowledge of sets to diagnose and predict Ebola Virus Disease (EVD). Ran and colleagues [82] applied the knowledge of fuzzy sets to intelligently select suppliers of medical consumption-related products. All these systems lack features for indigenous multilingual languages and voice enablement; they do not support HIV-comorbidity management, nor can they adequately assist West African medical personnel in managing HIV cases and patients in rural and suburban local communities. In this paper, we present the West African HIV and HIV-comorbidity Fuzzy Logic-based Multilingual Indigenous Diagnostic Software (WAHMIDS) and WAHMIMA (West African HIV Multilingual Informatics Mobile Application). Our work is novel as it is one of the first attempts to develop a voice-based multilingual HIV and HIV-comorbidity diagnostic and predictive system in the English language and selected indigenous West African languages (Yoruba, Igbo, and Hausa), together with its corresponding mobile app. Our system’s incorporation of indigenous mechanisms revolutionizes the field of medical software development. WAHMIDS will be a valuable and complementary resource for doctors and medical personnel working with HIV, HIV-comorbidity patients, and disease management in rural West African communities. WAHMIDS has the potential to be able to predict HIV severity in HIV patients, suggest suitable medication, and provide insight to effectively manage HIV and HIV comorbidity patients. It will help overcome language barriers, promote understanding, and enable efficient diagnosis. WAHMIMA (West African HIV Multilingual Informatics Mobile Application) will be a very useful tool for indigenous dwellers and English-speaking people. We outline the structure of this paper as follows: Section 2 covers the materials and methods, while Section 3 presents the results, demonstrations, and examples. Section 4 focuses on discussion, while Section 5 serves as the concluding section.

## 2. Materials and methods

### 2.1. Experimental dataset

Experimental data for this research was obtained through an additional extensive search of scientific literature. These sources provided information on symptomatic data associated with HIV patients. Please refer to S1 Appendix and S2 Table for further details. A total of over 72 HIV symptoms were incorporated into WAHMIDS software, with 29 for males and over 43 symptoms for females, especially because of pregnancy-related scenarios. HIV symptoms include weight loss, nervousness, headache, memory loss, depression, and dizziness [83–87]. These symptoms can be categorized into three different stages of HIV: the Acute HIV stage, the chronic stage (clinical latency), and the advanced stages (AIDS phase) of HIV [88]. WAHMIDs also has a functional section for HIV-comorbidities. Kindly refer to the supplementary files and S2 Table. S2 Table contains sample HIV data extracted from various scientific literature. S3 Table also depicts the translated sample HIV symptoms in Yoruba, Igbo, and Hausa languages. Some of these HIV symptoms were also utilized in the development of the West African HIV Multilingual Informatics Mobile Application (WAHMIMA). The translation of English language HIV symptoms into Yoruba, Igbo, and Hausa languages was obtained using online translators such as Google Translate [89] (https://translate.google.com) and Microsoft Translator [90] (https://www.bing.com/translator). Yoruba-speaking individuals helped to verify the correctness of the translations. The summarized information from the online translators for these indigenous languages can be found in Appendix 1.

### 2.2. Architecture of HIV informatics multilingual indigenous diagnostic and predictive systems for HIV management towards data-centric health interventions

**Fig 1:**
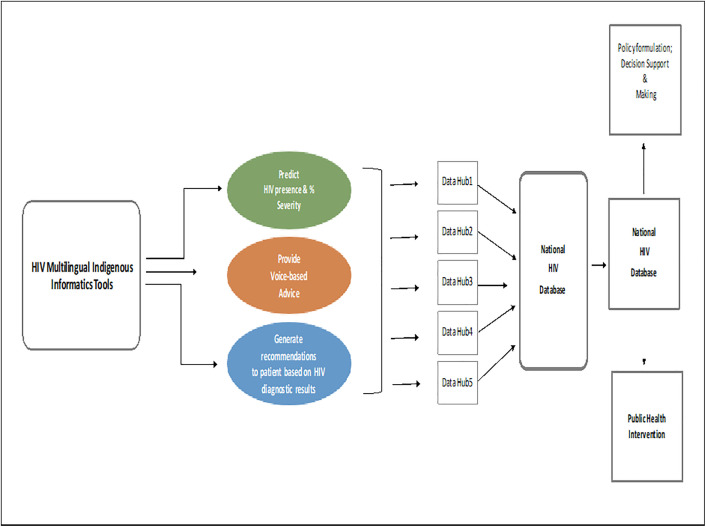
Architecture of Multilingual Informatics Indigenous Diagnostic and Predictive Systems for HIV Management Towards Data-Centric Health Interventions among West African Rural and Suburban Communities. This architecture depicts possible future applications of WAHMIDS software and WAHMIMA mobile application. It shows how these informatics tools can be applied for HIV management towards achieving equitable health interventions in the West African sub-region. It shows the predictive, voice-based advice and recommendation capabilities of the tools. It shows how the outcomes of the tools can be deployed into data hubs for onward transmission into the national database for the purpose of policy formulations and public health interventions.

The proposed architecture in Fig 1 represents the depiction of the application of WAHMIDS software and WAHMIMA mobile application (HIV multilingual indigenous informatics tools) towards data-centric health interventions among west African rural and suburban communities. WAHMIDS and WAHMIMA have voice-based capabilities in English and three other west African indigenous languages and knowledge about HIV. WAHMIDS can provide HIV diagnoses, recommendations, and prescriptions. The proposed architecture consists of different components, including the proposed deployment of WAHMIDS software to various rural and urban communities. Data obtained from and generated by these informatics tools will flow into the data hubs. The proposed data hubs (datahub1 to datahub5) consist of proposed hubs that will provide robust databases for storing anonymous, non-personal demographic health data collected from the usage of the software. Users of the software will be informed for the purpose of seeking the user’s consent and how the anonymous data collected will be utilized. Anonymous, non-personal demographic health data that will be collected from different health facilities will be stored at these hubs and will be subsequently transferred to the National HIV database in a secure manner. From the data hubs, information and data will flow into the national HIV database. The next stage is the flow of data from the national HIV database to the national ministry of health. The national ministry of health will continually utilize these anonymous data and engage statisticians and other researchers to analyze them for policy formulation, decision support, and quality public health interventions that will contribute positively to the well-being of rural and urban dwellers.

### 2.3. Description of the contents of the Schematic diagram in figure 3 and the description of the algorithm for Text-to-Speech Conversion in WAHMIDS

**Fig 2:**
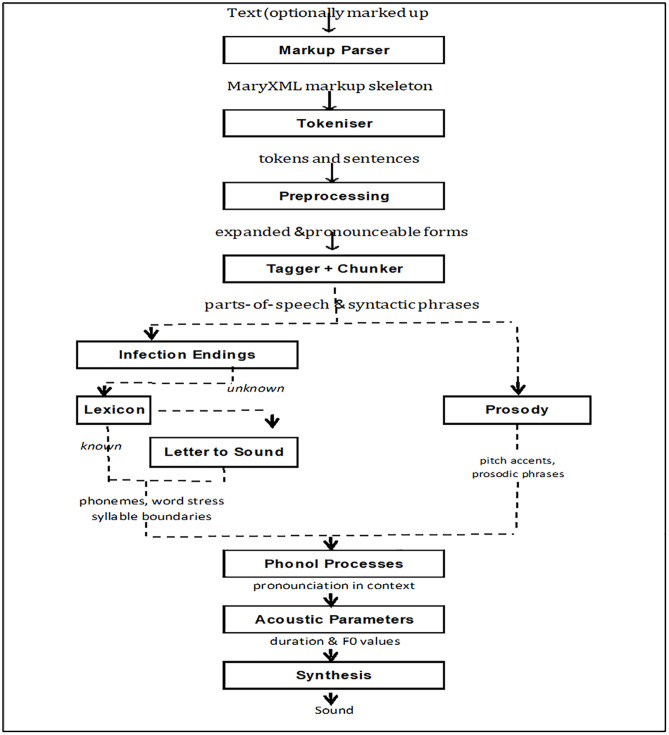
Schematic diagram depicting the Algorithm for text to speech conversion of Yoruba, Hausa, Igbo, and English language within WAHMIDS. This algorithm depicted in a flowchart-like representation helps to translate text to speech in the four languages within WAHMIDS software. Source: Adapted from [91] Schröder, M., and Trouvain, J, 2003).

The schematic diagram in Fig 2 depicts the process of the conversion of text to speech of the indigenous languages we integrated into WAHMIDS. The flowchart starts with the input of the indigenous language text into the parser. The text is processed by the parser and then sent to the tokenizer. The tokenizer breaks the texts into tokens and sentences. After the tokenizer phase, the tokens and sentences are sent to the preprocessing phase. In the pre-processing phase, the tokens and sentences are broken down into expandable and pronounceable forms. The expandable and pronounceable forms are then sent to the chunk and tagger phase for the purpose of syntactically classifying them into parts of speech and syntactic phrases. The parts of speech and the syntactic phrases are then made to pass through two separate phases, namely the inflection endings and the prosody phases. The prosody phase accepts the parts of speech and the syntactic phrases and converts them into pitch accents and prosodic phrases. The infection endings phase processes the parts of speech and the syntactic phrases into unknown representations and sends them into the lexicon phase. In the lexicon phase, the unknown representations are processed into known representations and sent to the letter-to-sound phase, where they are converted into phonemes, word stress syllable boundaries format, and then combined with pitch accents and periodic phrases and fed into the phonological processes phase. The phonological processes phase processed pronunciation and context of the input into it. The output from the phonological processes phase is sent as input into the acoustic parameter phase, which is later sent to the synthesis phase. The synthesis phase produces the sound of the text as the final output. The description of the algorithm for text-to-speech conversion for WAHMIDS is depicted in section 2.4.2. The flowchart for the algorithm that converts text to speech within WAHMIDS is depicted in Fig 2.

**Fig 3:**
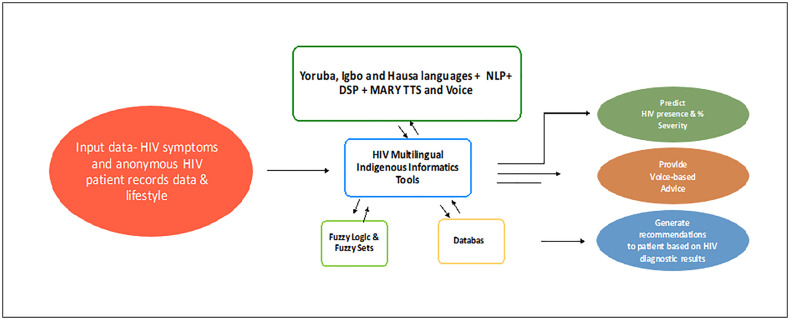
West African HIV Informatics Multilingual Indigenous Diagnostic and Predictive Software. This is the schematic depiction and description of WAHMIDS software.

### 2.4 Algorithms for WAHMIDS

#### 2.4.1 WAHMIDS’s algorithm for HIV diagnosis.

WAHMIDS’s algorithm for HIV diagnosis is depicted below (from step 1 to step 11).

***Begin***

**Step 1:** Input patient symptoms, *P*_*S1*_*, P*_*S2*_*, P*_*S3*_*, P*_*S4*_*, …, P*_*Sx*_*,* where x represents number of symptoms.

**Step 2:** Implement algorithm for Converting input from text-to-speech

**Step 3:** Assign weighing factors *(Wef*) to provide description for symptoms: Wef = 1 (mild HIV symptoms); *Wef* = 2 (moderate HIV symptoms); *Wef* = 3 (severe HIV symptoms).

**Step 4:** Weighing factors (*Wef*) of symptoms should undergo fuzzy rules processing.

**Step 5:** Compute the Degree of Membership through the comparison of Weighing factors (*Wef*) by mapping with fuzzy inputs.

This model: $E=\{\left(q_{i},\mu_{E}\left(q_{i}\right)|q_{i}\in H,\mu_{E}\left(q_{i}\right)\in [\text{0},\text{1}]\right\}$, helps confirm membership degree. A detailed description of E is in equation 1.

**Step 6:** Compute the rule base.

**Step 7**: Determining the execution strength of each rule.

**Step 8:** Compute the degree of truth of each rule through a non-zero minimum evaluation.

**Step 8+ (OPTIONAL): Specify if HIV patient has existing HIV-comorbidities and this influences the medication to be administered to such patients.**

**Step 9:** Calculate the total HIV severity present in a patient’s body by focusing on symptoms keyed-in and other closely related factors. This model was used to perform defuzzification.

Defuzzification model = $\frac{\sum_{i=\text{1}}^{m}\mu Q(q_{i})q_{i}}{\sum_{i=\text{1}}^{m}\mu Q(q_{i})}$, is essential to calculate the total HIV severity in a patient’s body and produce a result for the diagnosis (See Equation 4 that depicts the model description.)

**Step 10:** Produce an output for the diagnosis after various computations.

**Step 11:** Step 2; produce output for the diagnosis in Yoruba, Igbo, Hausa, and English languages.

***End***

#### 2.4.2 Algorithm for Converting input from text-to-speech within WAHMID.

The algorithm for converting input from text-to-speech within WAHMID is depicted below (from Step 1 to Step 10).

***Begin***

**Step 1:** Input the text in (Yoruba, Igbo, Hausa, and English language) into the Markup Parser.

**Step 2:** Tokenisation of the text into tokens and sentences through the MARY XML markup skeleton into the tokenizer.

**Step 3:** Tokens and sentences are subjected to pre-processing

**Step 4:** Input expanded……by implementing Tagger and Chunker

**Step 5:** Parts of speech and syntactic phrases are inputted into the Inflection Endings.

**Step 5.1** Lexicon

**Step 5.2** Letter to sound mapping

**Step 6:** process parts-of-speech and syntactic phrases by implementing Prosody.

**Step 7:** Implement Phonol processes.

**Step 8:** Implement Acoustic Parameters.

**Step 9:** Perform Synthesis of sound.

**Step 10:** Produce the Sound wave form of output.

***End***

The algorithm to convert text to speech within WAHMIDS is depicted in Fig 2.

### 2.5. Description of WAHMIDS’s algorithm for HIV diagnosis

The support for the internal mechanism of WAHMIDS is the integration of a database, knowledge base, and fuzzification engine based on the knowledge of fuzzy logic and fuzzy sets. The process of fuzzification caters for the conversion of input values emanating from each variable into a fuzzy representation from a specific set. Here, the specific set is depicted as {mild, moderate, severe}. The specific set’s components span over the variables. A fuzzy value range of “0.1 ≤ k < 0.3” defines the class of “mild HIV symptoms”; The fuzzy value range of “0.3 ≤ k < 0.6” depicts the class of “moderate HIV symptoms”; and the linguistic variable and class of “severe HIV symptoms” is represented by the fuzzy value range of “0.6 ≤ k < 0.8.” The first step in WAHMIDS’s algorithm for HIV diagnosis is inputting HIV symptoms. The second step centers around implementing an algorithm for converting input from text-to-speech (See section 3.4.1). The third step is the assignment of Weighing factors (Wef) to each variable to represent each HIV symptom’s severity. The fourth step subjects the weighing factors (Wef) of HIV symptoms to processing by fuzzy rules. The fifth step involves the computation of the membership degrees by comparing Weighing factors (Wef) and mapping them with the fuzzy inputs. The sixth step involves the calculation of the rule base. This phase is followed by determining each rule’s strength of execution in step seven. Step eight computes the degree of truth for each rule through a nonzero minimum evaluation. Step nine involves the computation of the total HIV severity in a patient’s body. Output for the diagnosis is captured in step ten. Step eleven produces the diagnosis output in local indigenous languages such as Yoruba, Igbo, Hausa, and English.

In the third step, fuzzy rules establish themselves. WAHMIDS contains an IF-THEN rule-based feature for different sets. Rule execution depends on whether a symptom is categorized as mild, moderate, or severe. If the outcome is TRUE, then the rule is executed. However, if the outcome is FALSE, then no rule is executed.

The degree of membership is determined in step four:


$$E=\left\{\left(q_{i},\mu_{E}\left(q_{i}\right)|q_{i}\in H,\mu_{E}\left(q_{i}\right)\in [\text{0},\text{1}]\right)\right\},$$ (1)

where *E* represents the fuzzy set, $q_{i}$ represents the HIV diagnostic variables, $\mu_{E}$ represents membership degree of $q_{i}$ in *E*, *H* is the set which contains the HIV diagnosis variables denoted by $q_{i}$.

Fuzzification provides a description for events that generate a fuzzy equivalent based on their real scalar value. This process generates a membership degree. To achieve fuzzification, one can apply various fuzzifiers. A triangular fuzzifier was utilized in this research:


$$\begin{array}{c} \mu_{E}(q_{i})=\left\{ \begin{array}{c} @l\text{1}\;\;\;\;\;\;\;\;\;\;\;\;\;\;\;\;\;if\;q_{i}<r\\ \frac{q_{i}-r}{s-r}\;\;\;\;\;\;\;\;if\;r\leq q_{i}<s\\ \frac{t-q_{i}}{t-s}\;\;\;\;\;\;\;\;if\;s\leq q_{i}<t\\ \text{0}\;\;\;\;\;\;\;\;\;\;\;\;\;\;\;\;if\;t<q_{i} \end{array}\right. \end{array}$$ (2)

where *r*, *s*, and *t* are parameters that impact on the shape of the triangle. Practically, we have *r*=0.1, *s*=0.2, *t*=0.3 representing mild symptoms; *r*=0.3, *s*=0.45, *t*=0.6 representing moderate symptoms; *r*=0.6, *s*=0.7, *t*=0.8 representing severe symptoms.

The fifth step involves the computation of the membership degrees by comparing Weighing factors (Wef) and mapping them with the fuzzy inputs. The sixth step involves the calculation of the rule base. The fuzzy inference engine contains a decision-making process-logic. The Root Sum Square (RSS) plays a major role by combining the outputs of the executed rules to produce useful inferences.

The ROOT-SUM-SQUARE (RSS) was utilized to combine overall effects of rules that apply. Its instrumental in computing the “fuzzy” centroid of the composite area. The Rule Base supplies set of input to the RSS. Such inputs are processed by the RSS to predict HIV levels inherent in a HIV patient.

RSS method integrates the effects of the important rules. It scales the functions of the significant rules at their respective magnitude. Finally, for the composite area, there is the construction of the fuzzy centroid. Our motivation for this choice was informed because, by design, the best weighted influence is provided to all rules that are being fired or performed. The equation for RSS is:


$$RSS=\sum {\mathrm{\backslash nolimits}}_{z=\text{1}}^{n}R_{z}^{\text{2}}$$ (3)

where **R**_**Z**_ is a fired rule or executed rules; and **z** is the strength of different rules and helps identify executed rules; the parameter **z = 1, 2, 3,…,n** is the number of fired rules for a given HIV diagnosis task.

The seventh step is to determine each rule’s degree of truth, through non-zero minimum evaluation. The eight step is to compute the possible level of HIV presence in a patient’s body based on the symptoms keyed-in. For defuzzification in the informatics system, we adopted the center of gravity (CoG) or Centroid of Area (CoA):


$$CoA=\frac{\sum_{i=\text{1}}^{m}\mu Q(q_{i})q_{i}}{\sum_{i=\text{1}}^{m}\mu Q(q_{i})}$$ (4)

where $\mu Q(q_{i})$ is the degree of *i* in a membership function, while $q_{i}$ represents the center value in the function. m, is the maximum value that *i* can achieve, *i* is the number of partitioned portions within the Centroid of Area (CoA). This methodology provides more computational flexibility and intuitive plausibility, hence informing our choice.

The ninth step was implemented and incorporated text-to-speech mechanism into WAHMIDS software. We adopted and included speech synthesis aspects into WAHMIDS, by adapting and including some text-to-speech java classes developed by MARY TTS. MARY stands for Modular Architecture for Research in sYynthesis [MARY Text-to-Speech System (MaryTTS). Some of the java classes from this modular architecture were integrated into our Java codes during the implementation of the system.

The algorithm’s tenth step outputs the results of the diagnosis based on the computations in previous steps. It transforms the results of the diagnosis from text-to-speech in English, by applying the voice computing mechanism to translate to Yoruba, Igbo, and Hausa languages.

**Some of the tables to support steps 1–10 within WAHMIDS algorithm are depicted in** **S4 Table** **to** **S9 Table. Other supporting tables can be found in** **S10 Table** **to** **S12 Table.**

### 2.6. Description of the algorithm for Text-to-Speech Conversion in WAHMIDS”

This is the description of the algorithm highlighted in section 2.4.2, which helps to convert text to speech. First, text in Yoruba, Igbo and Hausa indigenous languages are keyed into the Markup parser. The next stage is the tokenization of the texts into tokens and sentences through the MARY XML markup skeleton. This is followed by the pre-processing of the tokens and sentences. Next, the input is expanded when Tagger and Chunker are implemented on the pre-processed tokens and sentences. After this, syntactic phrases and parts of speech are inputted into the Inflection Endings and split into lexicon and letter to sound mapping. The next stage is the implementation of prosody, phonol processes and acoustic parameters. After these have been implemented, sound synthesis is performed and concluded with the production of sound wave from of the output of each indigenous word, and sentences.

## 3. Results

The result section consists of the depiction of the graphical user interfaces of the developed WAHMIDS software. It also consists of practical examples of the demonstrations of the WAHMIDS software, and the comparison of the outcome/predicted results of the WAHMIDS software with a manual computation of the algorithm depicted in the implementation of WAHMIDS. This section also presents the graphical user interface (GUI) of WAHMIMA mobile application.

### 3.1. Depiction of some of the GUI results of the WAHMIDS software and WAHMIMA mobile application

This section depicts the graphical user interfaces of WAHMIDS software and the WAHMIMA mobile application and contents. Some of the graphical user interface results of WAHMIDS software are presented in S2 Fig, S3 Figure, S4 Figure, S5 Figure, S6 Figure, S7 Figure, S8 Figure, S9 Figure, S10 Figure, S11 Figure, S12 Figure, S13 Figure, S14 Figure, S15 Figure, S16 Figure, S17 Figure, S18 Figure **in** S2 Appendix.

### 3.2. Demonstration and examples of WAHMIDS algorithm and software

In this section, we demonstrate mathematically how the WAHMIDS algorithm will work on sample HIV data obtained from existing scientific literature.

Certain paradigms are worth considering if HIV and HIV-comorbidity are to be effectively and efficiently managed. These include: (i) Early diagnosis and proper and effective management help to increase the lifespan of HIV patients. (ii) The transmission of HIV can be slowed down through the timely administering of Antiretroviral (ART/ARV) drugs coupled with advice and good counseling. (iii) A robust knowledge of HIV viral load within the human body or a corresponding metric will support medical personnel and clinicians in their quest to effectively and properly manage affected HIV patients. The significance of fuzzy algorithms cannot be overemphasized in these situations. In this section we demonstrate the significance of fuzzy algorithms in an HIV and HIV-comorbidity diagnostic multilingual informatics system by presenting some examples. Different scientific literature provided us with HIV symptoms **(See** S2 Table).

This example provides illustration for ten hypothetical West African HIV patients with different HIV symptoms as depicted in Table III. The patient’s IDs are: PAID1, PAID2, PAID3, PAID4, PAID5, PAID6, PAID7, PAID8, PAID9, and PAID10. **Ultimately, our focus will be on patient 8 (PAID8).** Each patient has any of the combinations of 25 HIV symptoms. The table also depicts the gender and HIV symptoms of the patients.

To better link section 3.1 to section 3.4, the description of the algorithms (in Section 3.4.1), will be carefully followed in this demonstration**. Here, in the first step of the WAHMIDS algorithm in section** **3.4.1**, the patients HIV symptoms **(*P***_***S1***_***, P***_***S2***_***, P***_***S3***_***, P***_***S4***_***, …, P***_***Sx***_**,),** where x represents number of symptoms, are input into the WAHMIDS algorithm.

The second step of the algorithm was the Implementation of another algorithm (the algorithm to convert text to speech within WAHMIDS is depicted in Fig 2.). The algorithm helps to convert input HIV symptoms from text-to-speech and translate the results and recommendations after final predictions from text-to-speech format.

The third step of WAHMIDs algorithm was to assign weighing factors *(Wef*) to provide description for symptoms. The severity of each HIV symptoms for each patient can be depicted by applying weighing factors(wf) to the Set **S**, where weighing factor of level **1 (**Wef = 1 (mild HIV symptoms); represents mild HIV symptoms, weighing factor of level **2** (*Wef* = 2 (moderate HIV symptoms);)represents moderate HIV symptoms, and weighing factor of level **3 (***Wef* = 3 (severe HIV symptoms) represents severe HIV symptoms. S4 Table, revealed the ten sample HIV patients exhibiting HIV symptoms but at different levels of severity. S5 Table depicted the different weighing factors allotted to each HIV symptoms of different HIV patients.

The fourth step of the WAHMIDS algorithm involved comparing respective weighing factors with fuzzy inputs through mapping. Here, weighing factors (Wef) of symptoms are made to undergo fuzzy rules processing. This activity was to help determine the degree of membership. For instance, if a patient told a medical doctor that he or she has a severe case of ulcer in the genitals, the doctor assesses the situation and confirms that the HIV symptom has a value of 3 (severe). In this case, the computation in the WAHMIDS software will compute the degree of the HIV symptom as follows: (3–1)/3 = 2/3 = 0.67. This result was achieved by applying the triangular fuzzifier to generate or produce triangular fuzzy numbers as shown in Table V. See Table V, for the generated triangular fuzzy numbers of the HIV symptoms for the different HIV patients. S6 Table depicts the computation of the derived triangular fuzzy values based on the values of the weighing factors of individual HIV symptom per HIV patient. Table V shows the derived triangular values for the HIV symptoms for our main **patient 8**, within WAHMIDS algorithm.

The fifth step of the WAHMIDs algorithm was to Compute the Degree of Membership through the comparison of Weighing factors (*Wef*) by mapping with fuzzy inputs, by utilizing the model E. This model: $E=\{\left(q_{i},\mu_{E}\left(q_{i}\right)|q_{i}\in H,\mu_{E}\left(q_{i}\right)\in [\text{0},\text{1}]\right\}$, assists to confirm the degree of membership. A detailed description of E can be found in equation 1.

S7 Table and S8 Table consist of the HIV symptoms for patient 8 with patient ID of (PAID8), the degree of HIV symptoms and the triangular fuzzy numbers (values) of the HIV symptoms. It also consists of HIV symptoms values keyed in for HIV patient 8 (ID = PAID8). S8 Table consists of HIV symptoms, degree of HIV symptoms, and the values of Triangular fuzzy numbers of the HIV symptoms for p HIV patient 8 (ID = PAID8).

The sixth step of WAHMIDs algorithm was to compute the rule base. S8 Table contains the Fuzzy rule base that has been defined. It has 24 rules and 25 HIV symptoms.

The seventh step was to determine the strength of execution of each rule. The seventh step of the WAHMIDS algorithm was the computation of the execution strength of rules of patient 8. See S10 Table, S11 Table and S12 Table. The set of rules that produced the non-zero minimum values were extracted; S12 Table consists of the Non-Zero Minimum Values for HIV–using 24 rules and 25 HIV symptoms for patient 8((PAID8), and others. Since our focus is on patient 8 ((PAID8), we identified the list of Rules that produced non-zero minimum values for patient 8 ((PAID8), These sets of rules are: Rules 1,2,3,4,5,6,7,8,9,10,11,12,13,14,15, 16, 17, 18, 19, 20, 21, 22, 23, 24. The following rules can be classified as: Mild = None; Moderate = R1, R9, R10, R11, R12, R19 and R23. Severe = R2, R3, R4, R5, R6, R7, R8, R13, R14, R15, R16, R17, R18, R20, R21, and R22 and R24. By applying the RSS equation from Equation 3, the execution strength of the rules was determined. Applying the RSS inference technique, we have:

$\;RSS=\sum_{z=\text{1}}^{n}R_{z}^{\text{2}}$ Equation 3,

Mathematically, these can be computed as follows:

For the class of Mild, $\sqrt{R_{\text{8}}^{\text{2}}+R_{\text{5}}^{\text{2}}+R_{\text{4}}^{\text{2}}+R_{\text{3}}^{\text{2}}+R_{\text{2}}^{\text{2}}+R_{\text{1}\text{1}}^{\text{2}}+R_{\text{1}\text{6}}^{\text{2}}+R_{\text{1}\text{7}}^{\text{2}}+R_{\text{1}\text{8}}^{\text{2}}++R_{\text{2}\text{0}}^{\text{2}}+R_{\text{1}\text{5}}^{\text{2}}}$

For the class of Mild = 0 =$\sqrt{{\text{0}}^{\text{2}}}=$ 0

For the moderate class, we have the followings: R1, R9, R10, R11, R12, R19 and R23 $=\sqrt{R_{\text{1}}^{\text{2}}+R_{\text{9}}^{\text{2}}+R_{\text{1}\text{2}}^{\text{2}}+R_{\text{1}\text{3}}^{\text{2}}+R_{\text{1}\text{4}}^{\text{2}}+R_{\text{1}\text{9}}^{\text{2}}+R_{\text{2}\text{3}}^{\text{2}}}$

$=\sqrt{{\text{0}.\text{33}}^{\text{2}}+{\text{0}.\text{33}}^{\text{2}}+{\text{0}.\text{33}}^{\text{2}}+{\text{0}.\text{33}}^{\text{2}}+{\text{0}.\text{33}}^{\text{2}}+{\text{0}.\text{33}}^{\text{2}}+{\text{0}.\text{33}}^{\text{2}}}=\;\sqrt{\text{0}.\text{7623}}=\;\text{0}.\text{87309793}$

For the severe class, we have the followings: = R6, R7, R10, R21, R22 and R24

$=\sqrt{R_{\text{6}}^{\text{2}}+R_{\text{7}}^{\text{2}}+R_{\text{10}}^{\text{2}}+R_{\text{21}}^{\text{2}}+R_{\text{22}}^{\text{2}}+R_{\text{24}}^{\text{2}}+}\;\sqrt{{\text{0}.\text{67}}^{\text{2}}+{\text{0}.\text{67}}^{\text{2}}+{\text{0}.\text{67}}^{\text{2}}+{\text{0}.\text{67}}_{\text{5}}^{\text{2}}+{\text{0}.\text{67}}^{\text{2}}+{\text{0}.\text{67}}^{\text{2}}}$

= $\sqrt{\text{0}.\text{4489}X\text{6}\;}=\sqrt{\text{2}..\text{6934}}=\;\text{1}.\text{641158}$

**WAHMIDS** algorithms in step eight helped to compute the degree of truth of each rule through a non-zero minimum evaluation. The eighth step of the WAHMIDs algorithm was to determine each rule’s degree of truth through non-zero minimum evaluation.

This implies

The execution strength for the mild category divided by the number of Rules for mild = 0/11 = 0

The execution strength for the moderate category divided by the number of Rules for moderate = 0.87309793/7 = 0.124728275714

The execution strength for the severe category divided by the number of Rules for severe = 1.641158/ 6 = 0.27352635461078

The step eight plus (Step 8+) is an optional step that helped to specify if the HIV patient has some possible HIV-comorbidities such as Hepatitis, Anaemia, Kidney problems, Insomnia, and this influences overall HIV severity.

**The ninth step of WAHMIDs algorithm was to compute** the overall severity of HIV present in an HIV patient’s body by focusing on symptoms keyed-in and other closely related factors. This model was used to perform defuzzification. The Defuzzification model = $\frac{\sum_{i=\text{1}}^{m}\mu Q(q_{i})q_{i}}{\sum_{i=\text{1}}^{m}\mu Q(q_{i})}$, is essential to calculate the total HIV severity in a patient’s body and produce a result for the diagnosis (See Equation 4 that depicts the model description.)

So, here, we shall be demonstrating how step nine of WAHMIDs algorithm will be applied to patient 8 with patient ID (PAID 8). Defuzzification process through a Centroid of Area (CoA) method, for patient 8 (PAID8), yielded 69.03% HIV severity as shown below:


$$\frac{\sum_{i=\text{1}}^{m}\mu Q(q_{i})q_{i}}{\sum_{i=\text{1}}^{m}\mu Q(q_{i})}=\;\frac{\left(\text{0}.\text{2}X\text{0}\;)+(\text{0}.\text{4}\text{5}\;X\text{0}.\text{1247})+(\text{0}.\text{8}X\text{0}.\text{2735}\right)}{\left(\text{0}+\text{0}.\text{1247}+\text{0}.\text{2735}\right)}=\;\frac{\text{0}+\;\text{0}.\text{0}\text{5}\text{6}\text{1}\text{1}\text{5}+\text{0}.\text{2}\text{1}\text{8}\text{8}}{\text{0}.\text{3}\text{9}\text{8}\text{2}}=\;\frac{\text{0}.\text{2}\text{7}\text{4}\text{9}}{\text{0}.\text{3}\text{9}\text{8}\text{2}}=\;\text{0}.\text{69035}$$

CoA = 0.69035

= 0.69035X 100 = 69.03% =** **CoA = 69.03% (falls into the severe category when compared with S13 Table).

The tenth step produced the output for the diagnosis after various computations. The eleventh step reverts to step two, to produce output for the diagnosis in Yoruba, Hausa, Igbo languages.

## 4. Discussion

This result is an estimated overall predicted percentage of HIV severity within a diagnosed patient’s (PAID8) body. This mathematical computation from WAHMID’s algorithm revealed a prediction of 69.03% HIV severity for the patient. In S10 Table, the interpretation of mathematical computation yielded a category of severe degree of HIV intensity (with a percentage range of 50–74%). The WAHMID’s software prediction for a similar patient produced a prediction of 72.32% HIV severity (as depicted in English, Yoruba, and Hausa languages in S3 Figure, S5 Figure, and S7 Figure in S2 Appendix, respectively).

The difference between the mathematical results and the software prediction was around +/- 3.29%; this disparity could be due to mathematical approximations inherent in mathematical computations, and it may also be due to other content (such as lifestyle questions) programmed into WAHMIDs software that wasn’t factored into the mathematical computation. Answers to lifestyle questions have an impact on the predicted results.

**In this section and subsequent sections, we discuss how our findings addressed the key research questions of this study as follows:**

(i) Medical personnel working in local West African communities can be aided in overcoming language communication barriers and supported in achieving effective HIV and HIV-comorbidity diagnosis and management by utilizing software like WAHMIDs. This software has been developed and is described in Section 4.1 and the mobile application WAHMIMA has been described in Section 4.2.
(ii) The Fuzzy logic, fuzzy sets, and Centroid of Area computational methods were explored and implemented in WAHMIDs so that the software will provide the needed support to complement the activities of English-speaking medical personnel in the effective diagnosis and management of HIV and HIV-comorbidity among indigenous West African rural, suburban, and urban communities.
(iii) The adoption and utilization of health informatics tools (WAHMIDS and WAHMIMA) by West African residents can help promote health management, diversity, equality, and inclusive care among affected populations. This has been described in Section 4.3.

### 4.1. WAHMIDS Software

A schematic depiction of the WAHMIDS software can be found in Fig 3. The software consists of a module/section for inputting HIV symptoms and selecting the severity level. It also includes a module/section that integrates indigenous language classes such as Yoruba, Igbo, and Hausa with MARY (Modular Architecture for Research in Synthesis) TTS (Text-to-Speech Synthesis) [92]. This integration is achieved through the application of Natural Language Processing (NLP) and Digital Signal Processing (DSP), along with voice integration. The informatics software interacts with fuzzy logic and fuzzy sets and stores data in a dedicated database within the software. The outputs of the software include the percentage (%) prediction of overall severity and possible stage of HIV, voice-based advice in different West African languages, and recommendations based on the HIV diagnostic results.

WAHMIDS software is a good region-specific HIV management software and specifically tailored to the West African region. It has multilingual features in three indigenous, widely spoken and accepted West African languages ((Yoruba, Hausa, and Igbo). WAHMIDS software is voice-enabled software. Multilingual Health informatics toolkits with voice-enabled features, for the management of HIV, are very scarce in West Africa. This tool will complement the efforts of medical personnel in sub-urban and rural communities of West Africa, in the effective management of HIV.

We hope WAHMID will assist in improving HIV diagnosis in rural communities of West Africa. This will help in reducing HIV transmissions. WAHMID is a novel HIV multilingual software in the West African sub-region to incorporate the diagnostics and predictive capabilities of Fuzzification and defuzzification [93], fuzzy sets [94–97] combined with text-to-speech mechanisms on selected West African indigenous popular languages.

### 4.2 West African HIV Multilingual Indigenous Mobile Application (WAHMIMA)

A mobile application was developed to complement medical efforts in rural and sub-urban regions of West Africa. We developed the West African HIV Multilingual Indigenous Mobile Application (WAHMIMA). It has the capability of creating awareness about HIV and how the transmissions can be reduced, in four different languages (English language, Yoruba, Hausa, and Igbo) languages, with voice-enabled capability. WAHMIMA (West African HIV Multilingual Informatics Mobile Application) is a useful region-specific HIV mobile application specifically targeted on the West African region. It is a voice-enabled mobile application that educates inhabitants about HIV symptoms, modes of transmissions, prevention methods in English, Yoruba, Hausa, and Igbo languages, which are languages commonly spoken in many inhabitants of West African subregion. Some of these languages are commonly spoken in some countries that around and bordering Nigeria such as Benin Republic, Togo, Ghana, Niger republic and Republic of Chad. The mobile application will create awareness to rural and suburban dwellers about HIV, factors responsible for the transmission of the disease, treatment advice for the disease. This mobile application will help to promote Equitable Artificial Intelligence (AI) in disease management. See S10 Figure **in** S2 Appendix.

### 4.3. A Catalyst for Promoting Equitable AI in Health care and management support to medical workers and HIV and HIV-comorbidity patients.

First, we anticipate that these informatics tools (WAHMIDS and WAHMIMA) can act as catalysts for promoting equitable AI in healthcare; that will help promote diversity, equity, and inclusion in public health management in many ways [95–99].

Second, the development and utilization of these informatics tools can act as catalysts that will help address public health disparities and racial disparities. This can help government pay more attention to low-health resource settings and regions with people that lack comprehensive healthcare facilities and resources—this will enable government to provide the needed public health manpower and health facilities in such regions by providing access to advanced diagnostic tools and expert knowledge, these tools can help reduce the gap in healthcare quality delivery amongst different regions and communities [100–105].

Third, creating and using this local multilingual informatics tool can help collect a wide range of anonymous patient information, including demographic and regional data, which is important for creating better and more inclusive healthcare plans that provide necessary resources and support for many local communities in managing HIV effectively. In our proposed system architecture for multilingual Indigenous diagnostic and predictive systems for managing HIV, many features can help gather various anonymous patient data. Further work will involve conducting region-specific user evaluation of the software for the four (4) languages in different countries within the West African sub-region. These health informatics tools will help in meeting the United Nations Sustainable Development Goals of goal number 3 (Good health and well-being) and goal number 10 (Reduced Inequality) [91,106] .

## 5. Conclusion

The knowledge of fuzzy logic, fuzzy sets, and voice technology was applied to develop WAHMIDS in three different popular indigenous West African languages, namely Yoruba, Hausa, and Igbo. This research aimed to establish a connection between indigenous African languages and the diagnosis and management of HIV disease. This is because communication and awareness are key determinants in combating diseases. The research also proposed a very useful architecture: a data-centric health intervention and multilingual Indigenous diagnostic and predictive systems for HIV management for the West African region of Africa. WAHMIDS will provide complementary support to health officers and medical doctors to help with the diagnosis of HIV in low-resource settings within communities that exist within the West African region. WAHMIMA was also developed to create more knowledge awareness about HIV among indigenous dwellers of different West African regions. The significance of these tools cannot be overemphasized. Utilization of this tool by medical and health workers and inhabitants of these regions will drastically reduce the time taken to diagnose and manage treatments for West African HIV patients and improve communication between health workers and patients. The overall effects of these are that there will be reduced rates of HIV transmissions and effective management of HIV patients. These improvements will lead to longevity among West African HIV infected patients, and the lives of the uninfected (unborn babies inclusive) will be protected and preserved. In the future, we aspire to evaluate WAHMIDS and WAHMIMA.

## Supporting information

S1 Table Table showing the synthesis of the 8 articles obtained from Systematic literature review. This table contains the 8 articles obtained from the systematic literature review. [64,65,67–72]
(DOC)

S2 Table WAHMIDS HIV Symptoms obtained from scientific literature. This table shows many HIV and HIV-comorbidity Symptoms of patients living with HIV sourced and obtained from many medical scientific manuscripts. [107–129]
(DOCX)

S3 Table Sample HIV Symptoms extracted from different scientific literature and depicted in Different prominent West African indigenous multilingual languages. This table shows selected HIV symptoms and some HIV-comorbidity conditions of patients living with HIV depicted in English language and different West African indigenous languages such as Yoruba, Hausa and Igbo languages.
(DOCX)

S4 Table Sample data from HIV Patients with 25 HIV symptoms. This table consists of 25 HIV symptoms for ten different HIV patients. The table also depicts information about the gender, ID number and the HIV symptoms of the patients.
(DOCX)

S5 Table Sample rating of the patients on HIV diagnosis variables. Sample of rating of the patients on HIV diagnosis variables. This table shows a sample rating of the patients on HIV diagnosis variables. This table shows the weights assigned to patients by doctors who have interacted with the patients concerned, as obtained from scientific literature.
(DOCX)

S6 Table Derived triangular values for HIV symptoms for patient 8, using WAHMIDS software. S6 Table shows the derived triangular values for the HIV symptoms for patient 8, within WAHMIDS software.
(DOCX)

S7 Table Values keyed in for HIV patient 8 (ID = PAID8). This table consists of HIV symptoms, degree of HIV symptoms, and the values of Triangular fuzzy numbers of the HIV symptoms.
(DOCX)

S8 Table HIV Symptoms for Patient, severity, and triangular fuzzy values of HIV symptoms. This table shows HIV symptoms for patients, corresponding severity, and triangular fuzzy values.
(DOCX)

S9 Table Fuzzy rule base for HIV–using 24 rules. This table consists of the defined Fuzzy rule base.
(DOCX)

S10 Table Details of the severities of 25 HIV symptoms and 24 rules. This table consists of the severities of 25 HIV symptoms and using 24 rules.
(DOCX)

S11 Table Equivalent details of the severity values of 25 HIV symptoms and using 24 rules. This table shows the details of the severity values of 25 HIV symptoms and using 24 rules.
(DOCX)

S12 Table Details of the Non-Zero Minimum Values for HIV–using 24 rules and 25 HIV symptoms reflecting the non-zero minimum values. This table consists of Non-Zero Minimum Values for HIV–using 24 rules and 25 HIV symptoms reflecting the non-zero minimum values.
(DOCX)

S13 Table Interpretation of predicted software result and mathematically computed algorithm result. This table contains the interpretation of the predictive software and the mathematical algorithm. Values depicted in the table were obtained from [88].
(DOCX)

S1 Appendix Multilingual version of HIV symptoms and HIV-comorbidity conditions, words, sentences used in WAHMIDS software. This file consists of the symptoms and interpretations of HIV and conditions of HIV-comorbidity used in WAHMIDS software including the English Words, Yoruba, Hausa and Igbo words used in WAHMIDS and their references.
(XLSX)

S2 Appendix **All Supplementary Figures used in the manuscript. S1 Figure**. PRISMA flow diagram of the identification process for the sample of 8 articles. The figure shows the systematic process of identifying the synthesized articles. This is S1 Figure_ Fig legend. **S2 Figure**. ENGLISH GUI of WAHMIDS software with sample HIV symptoms selection. This is the main English GUI of WAHMIDS. This green-colored Graphical User Interface (GUI) represents the English version of the West African HIV Multilingual Informatics Diagnostics and predictive Software. This is S2 Figure_ Fig legend. **S3 Figure**. English GUI of WAHMIDS software with HIV Predictive output and recommendation. This is the output of the English-version of the WAHMIDS software showing prediction, prescription, recommendation and advice. This is S3 Figure_ Fig legend. **S4 Figure.** YORUBA GUI of WAHMIDS software with sample HIV symptoms selection. This turquoise-colored Graphical User Interface (GUI) represents the Yoruba version of the West African HIV Multilingual Informatics Diagnostics and predictive Software. This is S4 Figure_ Fig legend. **S5 Figure**. YORUBA GUI of WAHMIDS software with HIV Predictive output and recommendation. This is the output of the Yoruba-version of the WAHMIDS software showing prediction, prescription, recommendation and advice. This is S5 Figure_ Fig legend. **S6 Figure.** Hausa GUI of WAHMIDS software with sample HIV symptoms selection. This yellow-colored Graphical User Interface (GUI) represents the Hausa version of the West African HIV Multilingual Informatics Diagnostics and predictive Software. This is Figure S6_ Fig legend. S7 Figure. Hausa GUI of WAHMIDS software with HIV Predictive output and recommendation. This is the output of the Hausa-version of the WAHMIDS software showing prediction, prescription, recommendation and advice. This is S7 Figure_ Fig legend. S8 Figure. Igbo Language GUI of WAHMIDS software with sample HIV symptoms selection. This pink-colored Graphical User Interface (GUI) represents the Hausa version of the West African HIV Multilingual Informatics Diagnostics and predictive Software. This is S8 Figure_ Fig legend. S9 Figure. ENGLISH GUI of WAHMIDS software with sample HIV and HIV-comorbidity real-conditions selections. This green-colored Graphical User Interface (GUI) represents the English version of the West African HIV Multilingual Informatics Diagnostics and predictive Software with sample HIV and HIV-comorbidity real-conditions selections. This is S9 Figure_ Fig legend. S10 Figure. West African HIV Multilingual Indigenous Mobile Application (WAHMIMA). This page is the home page of the WAHMIMA. The GUI consists of the logos of English language, Yoruba, Hausa, and Igbo languages. This is S10 Figure_ Fig legend. S11 Figure. West African HIV Multilingual Indigenous Mobile Application (WAHMIMA) English Page 1. This page is the output page of the WAHMIMA. The GUI displays the HIV/AIDS and HIV Symptoms in English language. The mobile application also has a voice-enabled feature that allows it to speak in English language. This is S11 Figure_ Fig legend. S14 Figure. West African HIV Multilingual Indigenous Mobile Application (WAHMIMA) Yoruba Page 2. This page is the output page of the WAHMIMA depicting transmission, prevention, diagnosis and treatment for HIV in Yoruba language in text and voice. The mobile application also has a voice-enabled feature that allows it to speak in Yoruba language. This is S14 Figure_ Fig legend. **S15 Figure**. West African HIV Multilingual Indigenous Mobile Application (WAHMIMA) Hausa Page 1. This page is the output page of the WAHMIMA. The GUI displays the HIV/AIDS and HIV Symptoms in Yoruba language. The mobile application also has a voice-enabled feature that allows it to speak in Yoruba language. This is S15 Figure_ Fig legend. **S16 Figure**. West African HIV Multilingual Indigenous Mobile Application (WAHMIMA) Hausa Page 2. This page is the output page of the WAHMIMA depicting transmission, prevention, diagnosis and treatment for HIV in Hausa language in text and voice. The mobile application also has a voice-enabled feature that allows it to speak in Hausa language. This is S16 Figure_ Fig legend. **S17 Figure**. West African HIV Multilingual Indigenous Mobile Application (WAHMIMA) Igbo Page 1. This page is the output page of the WAHMIMA. The GUI displays the HIV/AIDS and HIV Symptoms in Igbo language. The mobile application also has a voice-enabled feature that allows it to speak in Igbo language. This is S17 Figure_ Fig legend. **S18 Figure.** West African HIV Multilingual Indigenous Mobile Application (WAHMIMA) Igbo Page 2. This page is the output page of the WAHMIMA depicting transmission, prevention, diagnosis and treatment for HIV in Igbo language in text and voice. The mobile application also has a voice-enabled feature that allows it to speak in Igbo language. This is S18 Figure_ Fig legend.
(DOCX)
