# Supplementary material for: Multilingual voice-enabled informatics tools: Catalyst for equitable AI in HIV and HIV-comorbidity healthcare management
Source: PLoS One. 2025 Oct 21;20(10):e0332573. doi: 10.1371/journal.pone.0332573 (PMC12539699; doi:10.1371/journal.pone.0332573)
Supplement: S1 Table — This table contains the 8 articles obtained from the systematic literature review. [64,65,67–72] (DOC) [file pone.0332573.s001.doc]

**Systematic literature Synthesis for HIV Multilingual Informatics**

| **S/N** |  | **Aim/Methods** | **Advantages/**  **Contributions** | **Research gaps/Limitations** | **References** |
| --- | --- | --- | --- | --- | --- |
| **1** | **Expert System for the Intelligent Diagnosis of HIV/AIDS Using Fuzzy Cluster Means Algorithm.** | **a fuzzy cluster means algorithm was used to develop an intelligent diagnostic system for HIV/AIDs** | **an intelligent diagnostic system for HIV/AIDs** | **System lacked multilingual capabilities; lacked mobile application versions** | **Imianvan AA,** **Anosike UF, and Obi JC. Expert System for the Intelligent Diagnosis of HIV/AIDS Using Fuzzy Cluster Means Algorithm. Global Journal of Computer Science and Technology 2011; ISSN 0975-4172** |
| **2** | **MAVSCOT: A fuzzy logic-based HIV diagnostic system with indigenous multi-lingual interfaces for rural Africa** | **A fuzzy logic multilingual indigenous diagnostic system (MAVSCOT) was developed to address the management of HIV in rural South Africa** | **It was implemented to conduct diagnosis, provide recommendations and prescriptions. It was implemented in English language, and South African indigenous languages of Afrikaans, Zulu and IsiXhosa.** | **(1) One of the limitations of MAVSCOT is that the software can only be applied to the South African sub-region where the indigenous languages are spoken or understood. (2) MAVSCOT is that it did not fully address the issues of HIV-comorbidities.**  **(3) MAVSCOT lacked a mobile application version. (4) The indigenous languages in MAVSCOT cannot address the West African language barriers and challenges faced by HIV medical personnel within the West African sub-region. (5) MAVSCOT was not applied to effectively manage Hepatitis-HIV and other HIV comorbidity patients.** | **Oluwagbemi OO, Oluwagbemi FE, Jatto A, Hui C (2020) MAVSCOT: A fuzzy logic-based HIV diagnostic system with indigenous multi-lingual interfaces for rural Africa. PLoS ONE 15(11): e0241864. https://doi.org/10.1371/journal.pone.0241864** |
| **3** | **Human Immunodeficiency Virus (Hiv) Diagnosis Using Neuro-Fuzzy Expert System** | **It helped to develop an HIV diagnosis system that adopted elements of Fuzzy logic, fuzzy sets, and neural networks.** | **Their work created a model that helps to detect the risk levels** **of patients living with HIV. The results produced from their research revealed that the tool is user friendly and produces diagnosis results.** | **their informatics tool has fewer HIV symptoms** | **Ojeme BO, and Maureen A. Human Immunodeficiency Virus (Hiv) Diagnosis Using Neuro-Fuzzy Expert System. Oriental Journal of Computer Science and Technology 2014; 7(2)** |
| **4** | **HIVPCES: a WWW-based HIV patient care expert system** | **A WWW-based HIV patient care expert system was developed** | **The system adopts a methodology that integrates HTML and CGI-script for implementation** | **There was no mention of it being able to make comprehensive predictions. lacks voice-enabled features, they are not supportive of HIV-comorbidity management.** | **Atalay B, Potter WD, Haburchak D. HIVPCES: a WWW-based HIV patient care expert system, Proceedings of the 12th IEEE Symposium on Computer-Based Medical Systems (Cat. No.99CB36365), held between 18-20 June 1999, at Stamford, CT, USA, USA** |
| **5** | **Predictive Algorithm to Analyse Human Immunodeficiency Virus (HIV) Datasets in Medical Sector.** | **A Predictive Algorithm to Analyse Human Immunodeficiency Virus (HIV) Datasets in Medical Sector.** | **The predictive algorithm within their system can predict the possible percentage of HIV present in patients during prediction.** | **One of the limitations of their system is that it has few numbers of HIV symptoms integrated into the system.** | **Joglekar A, Lakshmi GP and Jani M.An Implementation of Predictive Algorithm to Analyse Human Immunodeficiency Virus (HIV) Datasets in Medical Sector. International Conference on Eco Friendly and Socially Responsive Economy and Equity: SIssues and Challenges of 21st Century for Emergent Sustainable Development amongst SAARC Countries 2017; 249-257** |
| **6** | **A mobile-based SMS system for the diagnosis of HIV infection** | **A mobile-based SMS system for the diagnosis of HIV infection amongst early infants in Zambia was developed.** | **Their mobile-based system has the capability of disseminating blood test results to laboratories by text messaging, where HIV screening takes place** | **It lacked multilingual capabilities.**  **lacks voice-enabled features, they are not supportive of HIV-comorbidity management.** | **Seidenberg P, Nicholson S, Schaefer M, Semrau K, Bweupe M, N, Bonawitz R, Chitembo L, Goggin C & Thea DM. Early infant diagnosis of HIV infection in Zambia through mobile phone texting of blood test results, Bulletin of the World Health Organization 2012;90:348-356** |
| **7** | **A novel AIDS/HIV intelligent medical consulting system based on expert systems** | **Developed a novel AIDS/HIV intelligent medical consulting system based on expert systems** | **Their system has the capability of providing consulting services based on available relevant input data** | **Lacked multilingual capabilities; lacked voice-enabled capabilities**  **lacks voice-enabled features, they are not supportive of HIV-comorbidity management.** | **Ebrahimi AP, Ashlaghi AT, and Rad MM. A novel AIDS/HIV intelligent medical consulting system based on expert systems, Journal of Educational and Health Promotion. 2013; 2: 54; doi: 10.4103/2277-9531.119041** |
| **8** | **Utility of an Interactive Voice Response System to Assess Antiretroviral Pharmacotherapy Adherence Among Substance Users Living with HIV/AIDS in the Rural South** | **An HIV Interactive telephone-based Voice Response self-monitoring system was developed** | **Their system was useful in assessing daily adherence of HIV anti-retroviral medication.** | **One of the limitations of their system was that it is not a diagnostic system and is not able to validate the expiration of HIV antiretroviral medication.**  **lacked indigenous multilingual languages features, lacks voice-enabled features.** | **Tucker JA, Simpson CA, Huang J, Roth DL, and Stewart KE. Utility of an Interactive Voice Response System to Assess Antiretroviral Pharmacotherapy Adherence Among Substance Users Living with HIV/AIDS in the Rural South, AIDS Patient Care STDS 2013; 27(5): 280–286; doi: 10.1089/apc.2012.0322.** |
|  |  |  |  |  |  |

**S1 Table. Table showing the synthesis of the 8 articles obtained from Systematic literature review .** This table contains the 8 articles obtained from the systematic literature review. This is the S1 Table legend
