# Supplementary material for: Multilingual voice-enabled informatics tools: Catalyst for equitable AI in HIV and HIV-comorbidity healthcare management
Source: PLoS One. 2025 Oct 21;20(10):e0332573. doi: 10.1371/journal.pone.0332573 (PMC12539699; doi:10.1371/journal.pone.0332573)
Supplement: S2 Table — This table shows many HIV and HIV-comorbidity Symptoms of patients living with HIV sourced and obtained from many medical scientific manuscripts. [107–129] (DOCX) [file pone.0332573.s002.docx]

| **HIV symptomatic Data** | **HIV and HIV-comorbidity Symptoms of patients living with HIV gotten from medical literature** | **Citations** |
| --- | --- | --- |
| Data Pack 1 | Depressive symptoms | [83]  [84]  [85]  [86]  [87]  [88]  [89] |
| Data Pack 2 | Weight Loss, Nausea/Vomiting, Poor sleep, Muscle aches/Joint pain, Fatigue, Anxiety/Nervous, Sadness, Numbness/Pain in the feet, Headache, Memory loss, Dizzy/Dizziness, Bloating/Abdominal pain, Poor appetite, Diarrhea, Loss of appetite, Rash, | [90]  [91]  [92]  [93] |
| Data Pack 3 | Sleep disorders, Fatigue, Fevers, Dizziness, Hand/foot pain, Memory loss, Nausea, Diarrhoea, Sadness, , Skin problems Cough, Headache, Sweats/chills, Weight loss, Appetite loss, Eye trouble, Chest pain, Abdominal pain, Muscle/joint pain; | [90]  [91]  [93]  [94]  [95] |
| Data Pack 4 | Symptoms frequently reported by People Living with HIV(PLWHIV):  Abdominal pain, Anxiety, Changes in body weight/fat, Cognitive decline, Diarrhoea, Fatigue, Headaches, Insomnia, Joint pain/stiffness, Loss of strength, Muscle pain, Nausea/vomiting, Peripheral neuropathy, | [110] |
| Data Pack 5 | Weakness, Poor appetite, Hot or cold spells, Shortness of breath, Faintness/dizziness, Pain in heart/chest | [91]  [93]  [96] |
| Data Pack 6 | Dementia | [97] |
| Data Pack 7 | Stress (associated with HIV) | [98]  [99] |
| Data Pack 8 | Anxiety and Depression | [83]  [89]  [93]  [100]  [101]  [102]  [103] [104]  [105]  [106]  [84]  [85] |
| Data Pack 9 | respiratory, digestive, and psychological symptoms, cognitive concerns, and skin disorders. | [109] |
| Data Pack 10 | pain, fatigue, low motivation, stigma, and unemployment were related to loneliness. Loneliness increased the odds of cognitive impairment, low mood, stress, and poor physical health | [98]  [107] |
| Data Pack 11 | Fatigue, Depression and Pain | [89]  [108]  [105]  [106]  [84]  [85]  [83] |

**S2 Table**: WAHMIDS HIV Symptoms obtained from scientific literatures.
