# Supplementary material for: Multilingual voice-enabled informatics tools: Catalyst for equitable AI in HIV and HIV-comorbidity healthcare management
Source: PLoS One. 2025 Oct 21;20(10):e0332573. doi: 10.1371/journal.pone.0332573 (PMC12539699; doi:10.1371/journal.pone.0332573)
Supplement: S3 Table — This table shows selected HIV symptoms and some HIV-comorbidity conditions of patients living with HIV depicted in English language and different West African indigenous languages such as Yoruba, Hausa and Igbo languages. (DOCX) [file pone.0332573.s003.docx]

| **S/N** | **Symptom Identifier** | **HIV Symptom in English Language** | **HIV Symptom in Yoruba Language** | **HIV Symptom in Hausa Language** | **HIV Symptom in Igbo Language** |
| --- | --- | --- | --- | --- | --- |
| 1 | (s1) | Abnormal swelling | Inu wiwu/Ipa wiwu | Ciki Kumburi | Abdom oziza |
| 2 | (s2) | Anxiety | Aniyan | Juyayi | Nchegbu |
| 3 | (s7) | Dementia | Iyawere/(Isonu iranti) | Gingin tsufa | Mgbaka |
| 4 | (s10) | Fatigue | Rire | Gajiya | Ike Ogwugwu |
| 5 | (s11) | Fever | Iba | zazzabi | Ahuhu |
| 6 | (s13) | Headache | Orififo | Ciwon kai | Isi mgbu |
| 7 | (s21) | Sexual dysfunction | Ibalopo Ailoye | Dawowa Kunnen | Mmekoahu  Mmekoahu |
| 8 | (s18) | Night sweats | Ooru ale | Rika Zufa Dare | Uwe mmiri awali |
| 9 | (s14) | Joint Pain (Rheumatism) | Lakuregbe | Da hadin gwiwa Zafi | nkwonkwo mgbu |
| 10 | (s16) | Muscle aches | Isan Irora | Baka Kaikayi | Akwara mgbu |
| 11 | (s26) | Ulcers in the Genitals | Ogbe lori awon abe | Rashin Lura | Onya afo na kenwe |
| 12 | (s29) | Weight loss | Isonu isonu | Aka ras lokutan | Ebufu ibu |
| 13 | (s36) | Abnormal vagina discharge | Ajeji Abe yosita | Mahaukaci farji sallama | Ndiiche mmamiri oruru |
| 14 | (s4) | Body Temperature | Ara otutu | Jici ciwo | Ahu opomoku |
| 15 | (s9) | Diarrhoea | Igbe gbuuruu | Cutar gudawa | Afo osisa |
| 16 | (s8) | Depression | Irewesi/ | Mawu yacin | Iduo |
| 17 | (s12) | Forgetfulness | Gbagbe | mantuwa | echefu |
| 18 | (s37) | Gonorrhoea | Gonoria | Kaba da ciwon sanyi | gonorrhea |
| 19 | (s33) | Heavy or Light periods | Wiwu tabi feerefee nkan osu/nkan asiko | Mutum Ko Lokaci | Aro ma o bu nihu oge |
| 20 | (s34) | Itching in the vaginal area | Nyun ni abe/obo | Acikin azzakari cikin farji | Itching na mpaghara ikpu |
| 21 | (s35) | Lower abdominal pain | Irora isale inu | Kanana cikin zafi | obere mgbu afọ |
| 22 | (s31) | Pain the upper right abdomen | Irora ni apa otun isale ikun | Pain a cikin Dama ciki | Mgbu n'elu aka nri afọ |
| 23 | (s32) | Missed periods | Nkan osu ti ko wa | Aka rasa lokutan | oge ịhụ nsọ agbaghara |
| 24 | (s38) | Painful intercourse | Irora ajosepo | M Ma’ amala | mmekọahụ na-egbu mgbu |
| 25 | (s39) | Painful Urination | Irora tito nigbagbogbo | Karkashin Karfin Kai | mmamịrị na-egbu mgbu |

**S3 Table. Sample HIV Symptoms extracted from different scientific literature and depicted in Different prominent West African indigenous multilingual languages**
