# Supplementary material for: Multilingual voice-enabled informatics tools: Catalyst for equitable AI in HIV and HIV-comorbidity healthcare management
Source: PLoS One. 2025 Oct 21;20(10):e0332573. doi: 10.1371/journal.pone.0332573 (PMC12539699; doi:10.1371/journal.pone.0332573)
Supplement: S4 Table — This table consists of 25 HIV symptoms for ten different HIV patients. The table also depicts information about the gender, ID number and the HIV symptoms of the patients. (DOCX) [file pone.0332573.s004.docx]

**S4 Table – West African (WA) HIV Multilingual Informatics**

Certain paradigms are worth considering if HIV is to be effectively and efficiently managed. These include: (i) Early diagnosis, proper and effective management helps to increase the lifespan of HIV patients. (ii) The transmission of HIV can be slowed down through timely administering of Antiretroviral (ART/ARV) drugs coupled with advice and good counselling. (iii) A robust knowledge of HIV viral load within the human body or a corresponding metric, will support medical personnel and clinicians in their quest to effectively and properly manage affected HIV patients. The significance of fuzzy algorithm can not be overemphasized in these situations. In this section we demonstrate this significance of fuzzy algorithm in an HIV diagnostic multilingual informatics system, by presenting some examples. Different scientific literature provided us with HIV symptoms (See Table S1).

This example provides illustration for ten hypothetical West African HIV patients with different HIV symptoms as depicted in Table 3. The patient’s IDs are: PAID1, PAID2, PAID3, PAID4, PAID5, PAID6, PAID7, PAID8, PAID9, and PAID10. **Ultimately, our focus will be on Patient 8 (PAID8).** Each patient has any of the combinations of the 25 HIV symptoms. The table also depicts the gender, and HIV symptoms of the patients.

**S4 Table – WA HIV Multilingual Informatics**

| **S/N** | **Patient’s ID** | **Gender** | **HIV Symptoms** |
| --- | --- | --- | --- |
| 1 | PAID1 | Male | Abnormal swelling  Anxiety  Dementia  Fatigue  Fever  Headache  Sexual dysfunction  Night sweats  Joint Pain (Rheumatism  Muscle aches  Ulcers in the Genitals  Weight loss  Abnormal vagina discharge  Body Temperature  Diarrhoea  Depression  Forgetfulness  Gonorrhoea  Heavy or Light periods  Itching in the vaginal area  Lower abdominal pain  Missed periods  Pain the upper right abdomen  Painful intercourse  Painful Urination  25 HIV Symptoms |
| 2 | PAID2 | Female |  |
| 3 | PAID3 | Male |  |
| 4 | PAID4 | Female |  |
| 5 | PAID5 | Female |  |
| 6 | PAID6 | Male |  |
| 7 | PAID7 | Male |  |
| **8** | **PAID8** | **Female** |  |
| 9 | PAID9 | Male |  |
| 10 | PAID10 | Female |  |

**S4 Table. Sample data from HIV Patients with 25 HIV symptoms**

This table consists of 25 HIV symptoms for ten different HIV patients. The table also depicts information about the gender, ID number and the HIV symptoms of the patients.
