# Supplementary material for: Multilingual voice-enabled informatics tools: Catalyst for equitable AI in HIV and HIV-comorbidity healthcare management
Source: PLoS One. 2025 Oct 21;20(10):e0332573. doi: 10.1371/journal.pone.0332573 (PMC12539699; doi:10.1371/journal.pone.0332573)
Supplement: S5 Table — Sample of rating of the patients on HIV diagnosis variables. This table shows a sample rating of the patients on HIV diagnosis variables. This table shows the weights assigned to patients by doctors who have interacted with the patients concerned, as obtained from scientific literature. (DOCX) [file pone.0332573.s005.docx]

To better link section 3.1 to section 3.4, the description of the algorithms will be carefully followed in this demonstration. From step 1 of the WAHMIDS software algorithm in section 3.4, the patients HIV symptoms are input into the WAHMIDS software. The severity of each HIV symptoms for each patient can be depicted by applying weighing factors(wf) to the Set **S**, where weighing factor of level **1** represents mild HIV symptom, weighing factor of level **2** represents moderate HIV symptom, and weighing factor of level **3** represents severe HIV symptom. **S4** **Table** revealed ten HIV patients exhibiting similar HIV symptoms but at different levels of severity. **S5 Table**  depicted the different weighing factors allotted to each HIV symptoms of different HIV patients.

**S5 Table. Depiction of weights allotted to patients HIV symptoms….by doctors who have interacted with the patients involved.**

| Patient ID | Abnormal swelling | Anxiety | Dementia | Fatigue | Fever | Headache | Sexual dysfunction | Night sweats | Joint Pain (Rheumatism | Muscle aches | Ulcers in the Genitals | Weight loss | Abnormal vagina discharge | Body Temperature | Diarrhoea | Depression | Forgetfulness | Gonorrhoea | Heavy or Light periods | Itching in the vaginal area | Lower abdominal pain | Missed periods | Pain the upper right abdomen | Painful intercourse | Painful Urination |
| --- | --- | --- | --- | --- | --- | --- | --- | --- | --- | --- | --- | --- | --- | --- | --- | --- | --- | --- | --- | --- | --- | --- | --- | --- | --- |
| PAID1 | 3 | 3 | 2 | 3 | 1 | 3 | 3 | 1 | 1 | 3 | 1 | 3 | 2 | 1 | 1 | 3 | 1 | 2 | 1 | 2 | 1 | 3 | 2 | 1 | 2 |
| PAID2 | 2 | 3 | 1 | 2 | 3 | 1 | 2 | 3 | 1 | 2 | 3 | 1 | 1 | 2 | 3 | 2 | 3 | 2 | 3 | 2 | 3 | 2 | 3 | 2 | 3 |
| PAID3 | 3 | 1 | 1 | 1 | 1 | 1 | 1 | 1 | 1 | 1 | 1 | 1 | 2 | 1 | 2 | 2 | 1 | 1 | 2 | 1 | 2 | 2 | 1 | 2 | 1 |
| PAID4 | 3 | 2 | 3 | 2 | 3 | 2 | 3 | 2 | 3 | 2 | 1 | 3 | 1 | 1 | 2 | 3 | 2 | 3 | 3 | 2 | 3 | 3 | 2 | 3 | 2 |
| PAID5 | 3 | 2 | 1 | 3 | 2 | 1 | 3 | 2 | 1 | 3 | 2 | 1 | 1 | 1 | 3 | 1 | 2 | 3 | 1 | 2 | 3 | 1 | 2 | 3 | 1 |
| PAID6 | 2 | 1 | 2 | 1 | 2 | 1 | 2 | 1 | 2 | 1 | 2 | 1 | 3 | 1 | 1 | 1 | 1 | 1 | 1 | 1 | 1 | 1 | 1 | 1 | 1 |
| PAID7 | 3 | 3 | 2 | 3 | 3 | 3 | 3 | 3 | 3 | 3 | 3 | 1 | 3 | 2 | 1 | 2 | 2 | 2 | 1 | 2 | 2 | 2 | 1 | 2 | 2 |
| **PAID8** | **3** | **3** | **3** | **3** | **3** | **3** | **3** | **3** | **3** | **3** | **3** | **3** | **3** | **3** | **3** | **3** | **3** | **3** | **3** | **3** | **3** | **3** | **3** | **3** | **3** |
| PAID9 | 2 | 2 | 2 | 2 | 2 | 2 | 2 | 2 | 2 | 2 | 2 | 2 | 2 | 2 | 2 | 2 | 2 | 2 | 2 | 2 | 2 | 2 | 2 | 2 | 2 |
| PAID10 | 3 | 2 | 3 | 2 | 2 | 3 | 2 | 3 | 2 | 3 | 2 | 3 | 2 | 3 | 2 | 3 | 2 | 3 | 2 | 3 | 2 | 3 | 2 | 3 | 2 |

This table shows a sample of rating of the patients on HIV diagnosis variables. This table shows the weights assigned to patients by doctors who have interacted with the patients concerned, as obtained from scientific literature.
