# Supplementary material for: Multilingual voice-enabled informatics tools: Catalyst for equitable AI in HIV and HIV-comorbidity healthcare management
Source: PLoS One. 2025 Oct 21;20(10):e0332573. doi: 10.1371/journal.pone.0332573 (PMC12539699; doi:10.1371/journal.pone.0332573)
Supplement: S6 Table — shows the derived triangular values for the HIV symptoms for patient 8, within WAHMIDS software. (DOCX) [file pone.0332573.s006.docx]

The third step of the WAHMIDS software’s algorithm applied fuzzy rules to weighing factors of HIV symptoms. S5 Table, depicts the computation of the derived triangular fuzzy values based on the values of the weighing factors of individual HIV symptom per HIV patient.

The fourth step of the WAHMIDS algorithm involves comparing respective weighing factors with fuzzy inputs, through mapping. This activity is to assist to determine the degree of membership. For instance, if a patient tells a medical doctor that he or she has a severe case of ulcer in the genitals, the doctor assesses the situation and confirms that the HIV symptom has a value of 3 (severe). In this case, the computation in the WAHMIDS software will compute the degree of the HIV symptom as follows: (3–1)/3 = 2/3 = 0.67. This result was achieved by applying the triangular fuzzifier to generate or produce triangular fuzzy numbers as shown in S6 Table. See **S6 Table**, for the generated triangular fuzzy numbers of the HIV symptoms for the different HIV patients.

**S6 Table. Derived triangular values for HIV symptoms for patient 8, using WAHMIDS software**

| Patient ID | Abnormal swelling | Anxiety | Dementia | Fatigue | Fever | Headache | Sexual dysfunction | Night sweats | Joint Pain (Rheumatism | Muscle aches | Ulcers in the Genitals | Weight loss | Abnormal vagina discharge | Body Temperature | Diarrhoea | Depression | Forgetfulness | Gonorrhoea | Heavy or Light periods | Itching in the vaginal area | Lower abdominal pain | Missed periods | Pain the upper right abdomen | Painful intercourse | Painful Urination |
| --- | --- | --- | --- | --- | --- | --- | --- | --- | --- | --- | --- | --- | --- | --- | --- | --- | --- | --- | --- | --- | --- | --- | --- | --- | --- |
|  |  |  |  |  |  |  |  |  |  |  |  |  | Weighing factor (Triangular Fuzzy value) | Weighing factor (Triangular Fuzzy value) | Weighing factor (Triangular Fuzzy value) |  |  |  |  |  |  |  |  |  |  |
| PAID1 | 0.67 | 0.67 | 0.33 | 0.67 | 0 | 0.67 | 0.67 | 0 | 0 | 0.67 | 0 | 0.67 | 2(2-1)/3= 0.33 | 1(0) | 1(0) | 0.67 | 0.33 | 0.67 | 0.33 | 0.67 | 0.33 | 0.67 | 0.33 | 0.33 | 0.67 |
| PAID2 | 0.33 | 0.67 | 0 | 0.33 | 0.67 | 0 | 0.33 | 0.67 | 0 | 0.33 | 0.67 | 0 | 1(1-1)/3 = 0 | 2(0.33) | 3 (0.67) | 2  0.33 | 3  0.67 | 2  0.33 | 3  0.67 | 2  0.33 | 3  0.67 | 2  0.33 | 3  0.67 | 2  0.33 | 3  0.67 |
| PAID3 | 0.67 | 0 | 0 | 0 | 0 | 0 | 0 | 0 | 0 | 0 | 0 | 0 | 2 (0.33) | 1(0) | 2(0.33) | 0.33 | 0 | 0 | 0.33 | 0 | 0.33 | 0.33 | 0 | 0.33 | 0 |
| PAID4 | 0.67 | 0.33 | 0.67 | 0.33 | 0.67 | 0.33 | 0.67 | 0.33 | 0.67 | 0.33 | 0 | 0.67 | 1 (0) | 1(0) | 2(0.33) | 0.67 | 0.33 | 0.67 | 0.67 | 0.33 | 0.67 | 0.67 | 0.33 | 0.67 | 0.33 |
| PAID5 | 0.67 | 0.33 | 0 | 0.67 | 0.33 | 0 | 0.67 | 0.33 | 0 | 0.67 | 0.33 | 0 | 1(0) | 1(0) | 3(0.67) | 0.33 | 0.67 | 0 | 0.33 | 0.67 | 0 | 0.33 | 0.67 | 0 | 0.33 |
| PAID6 | 0.33 | 0 | 0.33 | 0 | 0.33 | 0 | 0.33 | 0 | 0.33 | 0 | 0.33 | 0 | 3 (3-1)/3 = 0.67 | 1(0) | 1(0) | 0 | 0 | 0 | 0 | 0 | 0 | 0 | 0 | 0 | 0 |
| PAID7 | 0.67 | 0.67 | 0.33 | 0.67 | 0.67 | 0.67 | 0.67 | 0.67 | 0.67 | 0.67 | 0.67 | 0 | 3 (0.67) | 2(0.33) | 1(0) | 0.33 | 0.33 | 0.33 | 0 | 0.33 | 0.33 | 0.33 | 0 | 0.33 | 0.33 |
| **PAID8** | **0.67** | **0.67** | **0.67** | **0.67** | **0.67** | **0.67** | **0.67** | **0.67** | **0.67** | **0.67** | **0.67** | **0.67** | **3 (0.67)** | **3(0.67)** | **3(0.67)** | **0.67** | **0.67** | **0.67** | **0.67** | **0.67** | **0.67** | **0.67** | **0.67** | **0.67** | **0.67** |
| PAID9 | 0.33 | 0.33 | 0.33 | 0.33 | 0.33 | 0.33 | 0.33 | 0.33 | 0.33 | 0.33 | 0.33 | 0.33 | 2 (0.33) | 2(0.33) | 2(0.33) | 0.33 | 0.33 | 0.33 | 0.33 | 0.33 | 0.33 | 0.33 | 0.33 | 0.33 | 0.33 |
| PAID10 | 3 | 2 | 3 | 2 | 2 | 3 | 2 | 3 | 2 | 3 | 2 | 3 | 2 (0.67) | 3(0.67) | 2(0.33) | 0.67 | 0.67 | 0.33 | 0.67 | 0.33 | 0.67 | 0.33 | 0.67 | 0.33 | 0.67 |

S6 Table shows the derived triangular values for the HIV symptoms for patient 8, within WAHMIDS software.
