# Supplementary material for: Multilingual voice-enabled informatics tools: Catalyst for equitable AI in HIV and HIV-comorbidity healthcare management
Source: PLoS One. 2025 Oct 21;20(10):e0332573. doi: 10.1371/journal.pone.0332573 (PMC12539699; doi:10.1371/journal.pone.0332573)
Supplement: S7 Table — This table consists of HIV symptoms, degree of HIV symptoms, and the values of Triangular fuzzy numbers of the HIV symptoms. (DOCX) [file pone.0332573.s007.docx]

**S7 Table** consists of the HIV symptoms for patient 8 with patient ID of (PAID8), the degree of HIV symptoms and the triangular fuzzy numbers (values) of the HIV symptoms.

Values entered for patient 8 can be found in S7 Table as shown below:

S7 Table. Values keyed in for HIV patient 8 (ID = PAID8)

| **HIV Symptom number** | **HIV Symptoms for Patient (PAID8)** | **Degree of HIV symptom** | Triangular fuzzy numbers of the HIV symptoms |
| --- | --- | --- | --- |
| S1 | Abnormal swelling | Severe | 0.67 |
| S3 | Anxiety | Severe | 0.67 |
| S8 | Dementia | Severe | 0.67 |
|  | Fatigue | Severe | 0.67 |
|  | Fever | Severe | 0.67 |
|  | Headache | Severe | 0.67 |
| S30 | Sexual dysfunction | Severe | 0.67 |
|  | Night sweats | Severe | 0.67 |
| S18 | Joint Pain (Rheumatism) | Severe | 0.67 |
|  | Muscle aches | Severe | 0.67 |
| S36 | Ulcers in the Genitals | Severe | 0.67 |
| S39 | Weight loss | Severe | 0.67 |
| S2 | Abnormal vagina discharge | Severe | 0.67 |
|  | Body Temperature | Severe | 0.67 |
|  | Diarrhoea | Severe | 0.67 |
|  | Depression | Severe | 0.67 |
|  | Forgetfulness | Severe | 0.67 |
|  | Gonorrhoea | Severe | 0.67 |
|  | Heavy or Light periods | Severe | 0.67 |
|  | Itching in the vaginal area | Severe | 0.67 |
|  | Lower abdominal pain | Severe | 0.67 |
|  | Missed periods | Severe | 0.67 |
|  | Pain the upper right abdomen | Severe | 0.67 |
|  | Painful intercourse | Severe | 0.67 |
|  | Painful Urination | Severe | 0.67 |

S7 Table. Values keyed in for patient 8 (ID = PAID8)

This table consists of HIV symptoms, degree of HIV symptoms, and the values of Triangular fuzzy numbers of the HIV symptoms.
