# Supplementary material for: Multilingual voice-enabled informatics tools: Catalyst for equitable AI in HIV and HIV-comorbidity healthcare management
Source: PLoS One. 2025 Oct 21;20(10):e0332573. doi: 10.1371/journal.pone.0332573 (PMC12539699; doi:10.1371/journal.pone.0332573)
Supplement: S8 Table — This table shows HIV symptoms for patients, corresponding severity, and triangular fuzzy values. (DOCX) [file pone.0332573.s008.docx]

**S8 Table: HIV Symptoms for Patient, severity, and triangular fuzzy values of HIV symptoms**

|  |  |  |  |  |  |  |  |  |  |  |  |  |  |  |  |  |  |  |  |  |  |  |  |  |  |
| --- | --- | --- | --- | --- | --- | --- | --- | --- | --- | --- | --- | --- | --- | --- | --- | --- | --- | --- | --- | --- | --- | --- | --- | --- | --- |
| Symptoms | Abnormal swelling | Anxiety | Dementia | Fatigue | Fever | Headache | Sexual dysfunction | Night sweats | Joint Pain (Rheumatism | Muscle aches | Ulcers in the Genitals | Weight loss | Abnormal vagina discharge | Body Temperature | Diarrhoea | Depression | Forgetfulness | Gonorrhoea | Heavy or Light periods | Itching in the vaginal area | Lower abdominal pain | Missed periods | Pain the upper right abdomen | Painful intercourse | Painful Urination |
| Severity/Degree | Severe | Severe | Severe | Severe | Severe | Severe | Severe | Severe | Severe | Severe | Severe | Severe | Severe | Severe | Severe | Severe | Severe | severe | Severe | Severe | Severe | Severe | Severe | Severe | Mild |
| Triangular fuzzy numbers of the HIV symptoms | 0.67 | 0.67 | 0.67 | 0.67 | 0.67 | 0.67 | 0.67 | 0.67 | 0.6 7 | 0.67 | 0.67 | 0.67 | 0.67 | 0.67 | 0.67 | 0.67 | 0.67 | 0.67 | 0.67 | 0.33 | 0.67 | 0.67 | 0.67 | 0.67 | 0 |
