# Supplementary material for: Multilingual voice-enabled informatics tools: Catalyst for equitable AI in HIV and HIV-comorbidity healthcare management
Source: PLoS One. 2025 Oct 21;20(10):e0332573. doi: 10.1371/journal.pone.0332573 (PMC12539699; doi:10.1371/journal.pone.0332573)
Supplement: S9 Table — This table consists of the defined Fuzzy rule base. (DOCX) [file pone.0332573.s009.docx]

**[S9 Table.](https://journals.plos.org/plosone/article/file?type=supplementary&id=10.1371/journal.pone.0241864.s013)Fuzzy rule base for HIV–using 24 rules.**

This table consists of the defined Fuzzy rule base.

| Rule No. | Abnormal swelling | Anxiety | Dementia | Fatigue | Fever | Headache | Sexual dysfunction | Night sweats | Joint Pain (Rheumatism | Muscle aches | Ulcers in the Genitals | Weight loss | Abnormal vagina discharge | Body Temperature | Diarrhoea | Depression | Forgetfulness | Gonorrhoea | Heavy or Light periods | Itching in the vaginal area | Lower abdominal pain | Missed periods | Pain the upper right abdomen | Painful intercourse | Painful Urination |
| --- | --- | --- | --- | --- | --- | --- | --- | --- | --- | --- | --- | --- | --- | --- | --- | --- | --- | --- | --- | --- | --- | --- | --- | --- | --- |
| 1 | Mild | Moderate | Severe | Severe | Moderate | Severe | Moderate | Moderate | Severe | Moderate | Moderate | Moderate | Mild | Moderate | Severe | Severe | Moderate | severe | Mild | MOderate | Severe | mild | severe | moderate | moderate |
| 2 | Moderate | Moderate | Mild | Mild | Mild | Mild | Mild | Mild | Mild | Mild | Mild | Mild | Moderate | Moderate | Mild | Mild | Mild | severe | Mild | Mild | Mild | Mild | Mild | Mild | Mild |
| 3 | Mild | Mild | Mild | Mild | Mild | Mild | Mild | Mild | Mild | Mild | Mild | Mild | Mild | Mild | Mild | Mild | Mild | severe | Mild | Mild | Mild | Mild | Mild | Mild | Mild |
| 4 | Moderate | Mild | Mild | Mild | Mild | Mild | Severe | Severe | Severe | Severe | Severe | Severe | Moderate | Mild | Mild | Mild | Mild | severe | Mild | Severe | Severe | Severe | Severe | Severe | Severe |
| 5 | Mild | Severe | Mild | Severe | Mild | Severe | Mild | Severe | Mild | Mild | Mild | Mild | Mild | Severe | Mild | Severe | Mild | severe | Severe | Mild | Severe | Mild | Mild | Mild | Mild |
| 6 | Mild | Severe | Mild | Severe | Mild | Severe | Severe | Mild | Severe | Mild | Severe | Mild | Mild | Severe | Mild | Severe | Mild | severe | Severe | Severe | Mild | Severe | Mild | Severe | Mild |
| 7 | Severe | Mild | Severe | Mild | Severe | Mild | Severe | Mild | Severe | Severe | Severe | Severe | Severe | Mild | Severe | Mild | Severe | severe | Mild | Severe | Mild | Severe | Severe | Severe | Severe |
| 8 | Mild | Mild | Mild | Mild | Severe | Mild | Severe | Mild | Severe | Mild | Mild | Mild | Mild | Mild | Mild | Mild | Severe | severe | Mild | Severe | Mild | Severe | Mild | Mild | Mild |
| 9 | Moderate | Moderate | Moderate | Moderate | Moderate | Moderate | Moderate | Moderate | Moderate | Moderate | Moderate | Moderate | Moderate | Moderate | Moderate | Moderate | Moderate | severe | Moderate | Moderate | Moderate | Moderate | Moderate | Moderate | Moderate |
| 10 | Severe | Severe | Moderate | Severe | Severe | Severe | Severe | Severe | Severe | Severe | Severe | Mild | Severe | Severe | Moderate | Severe | Severe | severe | Severe | Severe | Severe | Severe | Severe | Severe | Mild |
| 11 | Mild | Mild | Severe | Severe | Mild | Mild | Severe | Severe | Mild | Mild | Severe | Mild | Mild | Mild | Severe | Severe | Mild | severe | Mild | Severe | Severe | Mild | Mild | Severe | Mild |
| 12 | Mild | Moderate | Mild | Moderate | Mild | Moderate | Mild | Moderate | Mild | Moderate | Mild | Moderate | Mild | Moderate | Mild | Moderate | Mild | severe | Moderate | Mild | Moderate | Mild | Moderate | Mild | Moderate |
| 13 | Mild | Mild | Mild | Mild | Mild | Moderate | Mild | Mild | Moderate | Mild | Mild | Mild | Mild | Mild | Mild | Mild | Mild | severe | Moderate | Mild | Mild | Moderate | Mild | Mild | Mild |
| 14 | Mild | Mild | Mild | Mild | Mild | Mild | Mild | Mild | Mild | Mild | **Moderate** | Mild | Mild | Mild | Mild | Mild | Mild | severe | Mild | Mild | Mild | Mild | Mild | **Moderate** | Mild |
| 15 | Mild | Moderate | Severe | Mild | Moderate | Severe | Mild | Moderate | Severe | Mild | Moderate | Severe | Mild | Moderate | Severe | Mild | Moderate | severe | Severe | Mild | Moderate | Severe | Mild | Moderate | Severe |
| 16 | Severe | Mild | Moderate | Severe | Mild | Moderate | Severe | Mild | Moderate | Severe | Mild | Moderate | Severe | Mild | Moderate | Severe | Mild | severe | Moderate | Severe | Mild | Moderate | Severe | Mild | Moderate |
| 17 | Moderate | Moderate | Moderate | Mild | Mild | Mild | Severe | Severe | Severe | Moderate | Moderate | Moderate | Moderate | Moderate | Moderate | Mild | Mild | severe | Mild | Severe | Severe | Severe | Moderate | Moderate | Moderate |
| 18 | Mild | Severe | Moderate | Mild | Severe | Moderate | Mild | Severe | Moderate | Mild | Severe | Moderate | Mild | Severe | Moderate | Mild | Severe | severe | Moderate | Mild | Severe | Moderate | Mild | Severe | Moderate |
| 19 | Moderate | Mild | Mild | Severe | Mild | Mild | Moderate | Mild | Mild | Severe | Mild | Mild | Moderate | Mild | Mild | Severe | Mild | severe | Mild | Moderate | Mild | Mild | Severe | Mild | Mild |
| 20 | Severe | Moderate | Moderate | Severe | Moderate | Moderate | Severe | Moderate | Moderate | Severe | Moderate | Moderate | Severe | Moderate | Moderate | Severe | Moderate | severe | Moderate | Severe | Moderate | Moderate | Severe | Moderate | Moderate |
| 21 | Mild | Severe | Severe | Moderate | Severe | Severe | Mild | Severe | Severe | Moderate | Severe | Severe | Mild | Severe | Severe | Moderate | Severe | severe | Severe | Mild | Severe | Severe | Moderate | Severe | Severe |
| 22 | Mild | Severe | Severe | Severe | Mild | Severe | Mild | Severe | Severe | Severe | Mild | Severe | Mild | Severe | Severe | Severe | Mild | severe | Mild | Severe | Severe | Severe | Mild | Mild | Severe |
| 23 | Moderate | Severe | Mild | Moderate | Severe | Mild | Moderate | Severe | Mild | Moderate | Severe | Mild | Moderate | Severe | Mild | Moderate | Severe | severe | Mild | Moderate | Severe | Mild | Moderate | Severe | Mild |
| 24 | Severe | Severe | Severe | Severe | Severe | Severe | Severe | Severe | Severe | Severe | Severe | Severe | Severe | Severe | Severe | Severe | Severe | severe | Severe | Severe | Severe | Severe | Severe | Severe | Severe |
