# Supplementary material for: Multilingual voice-enabled informatics tools: Catalyst for equitable AI in HIV and HIV-comorbidity healthcare management
Source: PLoS One. 2025 Oct 21;20(10):e0332573. doi: 10.1371/journal.pone.0332573 (PMC12539699; doi:10.1371/journal.pone.0332573)
Supplement: S10 Table — This table consists of the severities of 25 HIV symptoms and using 24 rules. (DOCX) [file pone.0332573.s010.docx]

**S12** [**Table .**](https://journals.plos.org/plosone/article/file?type=supplementary&id=10.1371/journal.pone.0241864.s013) **Non-Zero Minimum Values for HIV–using 24 rules and 25 HIV symptoms reflecting the non-zero minimum values**.

| Rule No. | **(s1)** Abnormal swelling | **(s2)** Anxiety | **(s7)** Dementia | **(s10)**Fatigue | **(s11)**Fever | **(s13)**Headache | **(s21)**Sexual dysfunction | **(s18)** Night sweats | **(s14)**  Joint Pain (Rheumatism | **(s16)**  Muscle aches | **(s26)**  Ulcers in the Genitals | **(s29)**  Weight loss | **(s36)**  Abnormal vagina discharge | **(s4)**  Body Temperature | **(s9)**  Diarrhoea | **(s8)**  Depression | **(s12)**  Forgetfulness | **(s37)**  Gonorrhoea | **(s33)**  Heavy or Light periods | **(s34)**  Itching in the vaginal area | **(s35)**  Lower abdominal pain | **(s32)**  Missed periods | **(s31)**  Pain the upper right abdomen | **(s38)**  Painful intercourse | **(s39)**  Painful Urination | **Conclusion**  **(C** ) | **Non-Zero Minimum Values**  **(N)** |
| --- | --- | --- | --- | --- | --- | --- | --- | --- | --- | --- | --- | --- | --- | --- | --- | --- | --- | --- | --- | --- | --- | --- | --- | --- | --- | --- | --- |
| 1 | - | - | 0.67 | 0.67 | - | 0.67 | - | - | 0.67 | - | - | - | - | - | 0.67 | 0.67 | - | 0.67 | 0 | 0.33 | 0.67 | 0 | 0.67 | - | - | Moderate | 0.33 |
| 2 | - | - | - | - | - | - | - | - | - | - | - | - | - | - | - | - | - | 0.67 | - | - | - | - | - | - | 0 | Severe | 0 |
| 3 | - | - | - | - | - | - | - | - | - | - | - | - | - | - | - | - | - | 0.67 | 0 | - | - | - | - | - | 0 | severe | **0** |
| 4 | - | - | - | - | - | - | 0.67 | 0.67 | 0.67 | 0.67 | 0.67 | 0.67 | - | 0 | 0 | 0 | 0 | 0.67 | 0 | - | 0.67 | 0.67 | 0.67 | 0.67 | - | severe | 0 |
| 5 | - | 0.67 | - | 0.67 | - | 0.67 | - | 0.67 | - | - | - | - | - | 0.67 | - | 0.67 | - | 0.67 | 0.67 | - | 0.67 | - | - | - | 0 | severe | 0 |
| 6 | - | 0.67 | - | 0.67 | - | 0.67 | 0.67 | - | 0.67 | - | 0.67 | - | - | 0.67 | - | 0.67 | - | 0.67 | 0.67 | - | - | 0.67 | - | 0.67 | 0 | severe | 0.67 |
| 7 | 0.67 | - | 0.67 | - | 0.67 | - | 0.67 | - | 0.67 | 0.67 | 0.67 | 0.67 | 0.67 | - | 0.67 | - | 0.67 | 0.67 | - | - | 0 | 0.67 | 0.67 | 0.67 | - | severe | 0.67 |
| **8** | **-** | **-** | **-** | **-** | **0.67** | **-** | **0.67** | **-** | **0.67** | **-** | **-** | **-** | **-** | **-** | **-** | **-** | **0.67** | **0.67** | **-** | - | - | 0.67 | - | - | - | severe | 0 |
| 9 | - | - | - | - | - | - | - | - | - | - | - | - | - | - | - | - | - | **0.67** | **0.33** | - | - | - | - | - | - | Moderate | **0.33** |
| 10 | **0.67** | **0.67** | **-** | **0.67** | **0.67** | **0.67** | **0.67** | **0.67** | **0.67** | **0.67** | **0.67** | **-** | **0.67** | **0.67** | **-** | **0.67** | **0.67** | **0.67** | **-** | **0.67** | **0.67** | **0.67** | **0.67** | **0.67** | **0** | Moderate | **0.67** |
| 11 | **-** | **-** | **0.67** | **0.67** | **-** | **-** | **0.67** | **0.67** | **-** | **-** | **0.67** | **-** | **-** | **-** | **0.67** | **0.67** | **-** | **0.67** | **-** | **0.67** | **0.67** | 0 | 0 | **0.67** | 0 | Moderate | **0** |
| 12 | **-** | **-** | **-** | **-** | **-** | **-** | **-** | **-** | **-** | **-** | **-** | **-** | **-** | **-** | **-** | **-** | **-** | **0.67** | **0.33** | **-** | **-** | **-** | **-** | **-** | **-** | Moderate | **0.33** |
| 13 | 0 | 0 | 0 | 0 | 0 | 0.33 | 0 | 0 | 0.33 | 0 | 0 | 0 | 0 | 0 | 0 | 0 | 0 | **0.67** | - | - | - | - | - | - | **0** | severe | **0.33/0** |
| 14 | 0 | 0 | 0 | 0 | 0 | 0 | 0 | 0 | 0 | 0 | 0.33 | 0 | 0 | 0 | 0 | 0 | 0 | **0.67** | - | - | - | - | - | - | - | severe | **0.33** |
| 15 | **-** | **-** | **0.67** | **0-** | **-** | **0.67** | **-** | **-** | **0.67** | **-** | **-** | **0.67** | **-** | **-** | **0.67** | **-** | **-** | **0.67** | **0.67** | **-** | **-** | **0.67** | **-** | **-** | **-** | **severe** | **0** |
| 16 | **0.67** | **-** | **-** | **0.67** | **-** | **-** | **0.67** | **-** | **-** | **0.67** | **-** | **-** | **0.67** | **-** | **-** | **0.67** | **-** | **0.67** | **-** | **-** | **0** | **-** | **0.67** | **-** | **-** | **severe** | **0** |
| 17 | **-** | **-** | **-** | **-** | **-** | **-** | **0.67** | **0.67** | **0.67** | **-** | **-** | **-** | **-** | **-** | **-** | **-** | **-** | **0.67** | **-** | - | 0.67 | 0.67 | - | - | - | **severe** | **0** |
| 18 | **0** | **0.67** | **-** | **0** | **0.67** | **-** | **-** | **0.67** | **-** | **-** | **0.67** | **-** | **-** | **0.67** | **-** | **-** | **0.67** | **0.67** | **-** | - | **0.67** | **-** | **-** | **0.67** | **-** | **severe** | **0** |
| 19 | **-** | **0** | **0** | **0.67** | **0** | **0** | **-** | **0** | **0** | **0.67** | **0** | **0** |  | **0** | **0** | **0.67** | **0** | **0.67** | **0** | **0.33** | **-** | **-** | **0.67** | **-** | **0** | **Moderate** | **0.33** |
| 20 | **0.67** | **-** | **-** | **0.67** | **-** | **-** | **0.67** | **-** | **-** | **0.67** | **-** | **-** | **0.67** | **-** | **-** | **0.67** | **-** | **0.67** | **-** | **-** | **-** | **-** | **0.67** | **-** | **-** | **severe** | **0** |
| 21 | **0** | **0.67** | **0.67** | **-** | **0.67** | **0.67** | **-** | **0.67** | **0.67** | **-** | **0.67** | **0.67** | **0** | **0.67** | **0.67** | **-** | **0.67** | **0.67** | **0.67** | **0** | **0.67** | **0.67** | **-** | **0.67** | **0.67** | **severe** | **0.67** |
| 22 | **-** | **0.67** | **0.67** | **0.67** | **-** | **0.67** | **-** | **0.67** | **0.67** | **0.67** | **-** | **0.67** | **-** | **0.67** | **0.67** | **0.67** | **-** | **0.67** | **0** | **-** | **0.67** | **0.67** | **0** | **0** | **-** | **severe** | **0.67** |
| 23 | **-** | **0.67** | **-** | **-** | **0.67** | **-** | **-** | **0.67** | **-** | **-** | **0.67** | **-** | **-** | **0.67** | **-** | **-** | **0.67** | **0.67** | **-** | **0.33** | **0.67** | **-** | **-** | **0.67** | **-** | **Moderate** | **0.33** |
| 24 | **0.67** | **0.67** | **0.67** | **0.67** | **0.67** | **0.67** | **0.67** | **0.67** | **0.67** | **0.67** | **0.67** | **0.67** | **0.67** | **0.67** | **0.67** | **0.67** | **0.67** | **0.67** | **0.67** | **-** | **0.67** | **0.67** | **0.67** | **0.67** | **-** | **severe** | **0.67** |
