# Supplementary material for: Multilingual voice-enabled informatics tools: Catalyst for equitable AI in HIV and HIV-comorbidity healthcare management
Source: PLoS One. 2025 Oct 21;20(10):e0332573. doi: 10.1371/journal.pone.0332573 (PMC12539699; doi:10.1371/journal.pone.0332573)
Supplement: S11 Table — This table shows the details of the severity values of 25 HIV symptoms and using 24 rules. (DOCX) [file pone.0332573.s011.docx]

**S11 Table. Equivalent details of the severity’s values of 25 HIV symptoms and using 24 rules**.

| Rule No. | Abnormal swelling | Anxiety | Dementia | Fatigue | Fever | Headache | Sexual dysfunction | Night sweats | Joint Pain (Rheumatism | Muscle aches | Ulcers in the Genitals | Weight loss | Abnormal vagina discharge | Body Temperature | Diarrhoea | Depression | Forgetfulness | Gonorrhoea | Heavy or Light periods | Itching in the vaginal area | Lower abdominal pain | Missed periods | Pain the upper right abdomen | Painful intercourse | Painful Urination |
| --- | --- | --- | --- | --- | --- | --- | --- | --- | --- | --- | --- | --- | --- | --- | --- | --- | --- | --- | --- | --- | --- | --- | --- | --- | --- |
| 1 | 0 | 0.33 | 0.67 | 0.67 | 0.33 | 0.67 | 0.33 | 0.33 | 0.67 | 0.33 | 0.33 | 0.33 | 0 | 0.33 | 0.67 | 0.67 | 0.33 | 0.67 | 0 | 0.33 | 0.67 | 0 | 0.67 | 0.33 | 0.33 |
| 2 | 0.33 | 0.33 | 0 | 0 | 0 | 0 | 0 | 0 | 0 | 0 | 0 | 0 | 0.33 | 0.33 | 0 | 0 | 0 | 0.67 | 0 | 0 | 0 | 0 | 0 | 0 | 0 |
| 3 | 0 | 0 | 0 | 0 | 0 | 0 | 0 | 0 | 0 | 0 | 0 | 0 | 0 | 0 | 0 | 0 | 0 | 0.67 | 0 | 0 | 0 | 0 | 0 | 0 | 0 |
| 4 | 0.33 | 0 | 0 | 0 | 0 | 0 | 0.67 | 0.67 | 0.67 | 0.67 | 0.67 | 0.67 | 0.33 | 0 | 0 | 0 | 0 | 0.67 | 0 | 0.67 | 0.67 | 0.67 | 0.67 | 0.67 | 0.67 |
| 5 | 0 | 0.67 | 0 | 0.67 | 0 | 0.67 | 0 | 0.67 | 0 | 0 | 0 | 0 | 0 | 0.67 | 0 | 0.67 | 0 | 0.67 | 0.67 | 0 | 0.67 | 0 | 0 | 0 | 0 |
| 6 | 0 | 0.67 | 0 | 0.67 | 0 | 0.67 | 0.67 | 0 | 0.67 | 0 | 0.67 | 0 | 0 | 0.67 | 0 | 0.67 | 0 | 0.67 | 0.67 | 0.67 | 0 | 0.67 | 0 | 0.67 | 0 |
| 7 | 0.67 | 0 | 0.67 | 0 | 0.67 | 0 | 0.67 | 0 | 0.67 | 0.67 | 0.67 | 0.67 | 0.67 | 0 | 0.67 | 0 | 0.67 | 0.67 | 0 | 0.67 | 0 | 0.67 | 0.67 | 0.67 | 0.67 |
| 8 | 0 | 0 | 0 | 0 | 0.67 | 0 | 0.67 | 0 | 0.67 | 0 | 0 | 0 | 0 | 0 | 0 | 0 | 0.67 | 0.67 | 0 | 0.67 | 0 | 0.67 | 0 | 0 | 0 |
| 9 | 0.33 | 0.33 | 0.33 | 0.33 | 0.33 | 0.33 | 0.33 | 0.33 | 0.33 | 0.33 | 0.33 | 0.33 | 0.33 | 0.33 | 0.33 | 0.33 | 0.33 | 0.67 | 0.33 | 0.33 | 0.33 | 0.33 | 0.33 | 0.33 | 0.33 |
| 10 | 0.67 | 0.67 | 0.33 | 0.67 | 0.67 | 0.67 | 0.67 | 0.67 | 0.67 | 0.67 | 0.67 | 0 | 0.67 | 0.67 | 0.33 | 0.67 | 0.67 | 0.67 | 0.67 | 0.67 | 0.67 | 0.67 | 0.67 | 0.67 | 0 |
| 11 | 0 | 0 | 0.67 | 0.67 | 0 | 0 | 0.67 | 0.67 | 0 | 0 | 0.67 | 0 | 0 | 0 | 0.67 | 0.67 | 0 | 0.67 | 0 | 0.67 | 0.67 | 0 | 0 | 0.67 | 0 |
| 12 | 0 | 0.33 | 0 | 0.33 | 0 | 0.33 | 0 | 0.33 | 0 | 0.33 | 0 | 0.33 | 0 | 0.33 | 0 | 0.33 | 0 | 0.67 | 0.33 | 0 | 0.33 | 0 | 0.33 | 0 | 0.33 |
| 13 | 0 | 0 | 0 | 0 | 0 | 0.33 | 0 | 0 | 0.33 | 0 | 0 | 0 | 0 | 0 | 0 | 0 | 0 | 0.67 | 0.33 | 0 | 0 | 0.33 | 0 | 0 | 0 |
| 14 | 0 | 0 | 0 | 0 | 0 | 0 | 0 | 0 | 0 | 0 | 0.33 | 0 | 0 | 0 | 0 | 0 | 0 | 0.67 | 0 | 0 | 0 | 0 | 0 | 0.33 | 0 |
| 15 | 0 | 0.33 | 0.67 | 0 | 0.33 | 0.67 | 0 | 0.33 | 0.67 | 0 | 0.33 | 0.67 | 0 | 0.33 | 0.67 | 0 | 0.33 | 0.67 | 0.67 | 0 | 0.33 | 0.67 | 0 | 0.33 | 0.67 |
| 16 | 0.67 | 0 | 0.33 | 0.67 | 0 | 0.33e | 0.67 | 0 | 0.33 | 0.67 | 0 | 0.33 | 0.67 | 0 | 0.33 | 0.67 | 0 | 0.67 | 0.33 | 0.67 | 0 | 0.33 | 0.67 | 0 | 0.33 |
| 17 | 0.33 | 0.33 | 0.33 | 0 | 0 | 0 | 0.67 | 0.67 | 0.67 | 0.33 | 0.33 | 0.33 | 0.33 | 0.33 | 0.33 | 0 | 0 | 0.67 | 0 | 0.67 | 0.67 | 0.67 | 0.33 | 0.33 | 0.33 |
| 18 | 0 | 0.67 | 0.33 | 0 | 0.67 | 0.33 | 0 | 0.67 | 0.33 | 0 | 0.67 | 0.33 | 0 | 0.67 | 0.33 | 0 | 0.67 | 0.67 | 0.33 | 0 | 0.67 | 0.33 | 0 | 0.67 | 0.33 |
| 19 | 0.33 | 0 | 0 | 0.67 | 0 | 0 | 0.33 | 0 | 0 | 0.67 | 0 | 0 | 0.33 | 0 | 0 | 0.67 | 0 | 0.67 | 0 | 0.33 | 0 | 0 | 0.67 | 0 | 0 |
| 20 | 0.67 | 0.33 | 0.33 | 0.67 | 0.33 | 0.33 | 0.67 | 0.33 | 0.33 | 0.67 | 0.33 | 0.33 | 0.67 | 0.33 | 0.33 | 0.67 | 0.33 | 0.67 | 0.33 | 0.67 | 0.33 | 0.33 | 0.67 | 0.33 | 0.33 |
| 21 | 0 | 0.67 | 0.67 | 0.33 | 0.67 | 0.67 | 0 | 0.67 | 0.67 | 0.33 | 0.67 | 0.67 | 0 | 0.67 | 0.67 | 0.33 | 0.67 | 0.67 | 0.67 | 0 | 0.67 | 0.67 | 0.33 | 0.67 | 0.67 |
| 22 | 0 | 0.67 | 0.67 | 0.67 | 0 | 0.67 | 0 | 0.67 | 0.67 | 0.67 | 0 | 0.67 | 0 | 0.67 | 0.67 | 0.67 | 0 | 0.67 | 0 | 0.67 | 0.67 | 0.67 | 0 | 0 | 0.67 |
| 23 | 0.33 | 0.67 | 0 | 0.33 | 0.67 | 0 | 0.33 | 0.67 | 0 | 0.33 | 0.67 | 0 | 0.33 | 0.67 | 0 | 0.33 | 0.67 | 0.67 | 0 | 0.33 | 0.67 | 0 | 0.33 | 0.67 | 0 |
| 24 | 0.67 | 0.67 | 0.67 | 0.67 | 0.67 | 0.67 | 0.67 | 0.67 | 0.67 | 0.67 | 0.67 | 0.67 | 0.67 | 0.67 | 0.67 | 0.67 | 0.67 | 0.67 | 0.67 | 0.67 | 0.67 | 0.67 | 0.67 | 0.67 | 0.67 |
