# Supplementary material for: Multilingual voice-enabled informatics tools: Catalyst for equitable AI in HIV and HIV-comorbidity healthcare management
Source: PLoS One. 2025 Oct 21;20(10):e0332573. doi: 10.1371/journal.pone.0332573 (PMC12539699; doi:10.1371/journal.pone.0332573)
Supplement: S13 Table — This table contains the interpretation of the predictive software and the mathematical algorithm. Values depicted in the table were obtained from [88]. (DOCX) [file pone.0332573.s013.docx]

| **Predicted Percentage Range** | **Degree of HIV intensity** | **Possible HIV Stage in the Medical Field** |
| --- | --- | --- |
| **0 - 24%** | **Mild** | **Acute HIV infection – sharp drop in concentration of cirulating CD+T cells** |
| **25% - 49%** | **Moderate** | **Chronic HIV infection(Clinical latency) CD4_T cells circulation – near normal, drop in viral loads.** |
| **50% - 74%** | **Severe** | **Chronic HIV infection(Clinical latency) CD4_T cells circulation – near normal, drop in viral loads.** |
| **75% - 100%** | **Very Severe** | **Full Blown AIDs ( drop in viral loads)/ Full -blown AIDS (most severe stage) – CD4 < 200 Cells/mm3** |

S13 Table: Interpretation of predicted software result and mathematically computed algorithm result.
