# Supplementary material for: Multilingual voice-enabled informatics tools: Catalyst for equitable AI in HIV and HIV-comorbidity healthcare management
Source: PLoS One. 2025 Oct 21;20(10):e0332573. doi: 10.1371/journal.pone.0332573 (PMC12539699; doi:10.1371/journal.pone.0332573)
Supplement: S2 Appendix — All Supplementary Figures used in the manuscript. S1 Figure. PRISMA flow diagram of the identification process for the sample of 8 articles. The figure shows the systematic process of identifying the synthesized articles. This is S1 Figure_ Fig legend. S2 Figure. ENGLISH GUI of WAHMIDS software with sample HIV symptoms selection. This is the main English GUI of WAHMIDS. This green-colored Graphical User Interface (GUI) represents the English version of the West African HIV Multilingual Informatics Diagnostics and predictive Software. This is S2 Figure_ Fig legend. S3 Figure. English GUI of WAHMIDS software with HIV Predictive output and recommendation. This is the output of the English-version of the WAHMIDS software showing prediction, prescription, recommendation and advice. This is S3 Figure_ Fig legend. S4 Figure. YORUBA GUI of WAHMIDS software with sample HIV symptoms selection. This turquoise-colored Graphical User Interface (GUI) represents the Yoruba version of the West African HIV Multilingual Informatics Diagnostics and predictive Software. This is S4 Figure_ Fig legend. S5 Figure. YORUBA GUI of WAHMIDS software with HIV Predictive output and recommendation. This is the output of the Yoruba-version of the WAHMIDS software showing prediction, prescription, recommendation and advice. This is S5 Figure_ Fig legend. S6 Figure. Hausa GUI of WAHMIDS software with sample HIV symptoms selection. This yellow-colored Graphical User Interface (GUI) represents the Hausa version of the West African HIV Multilingual Informatics Diagnostics and predictive Software. This is Figure S6_ Fig legend. S7 Figure. Hausa GUI of WAHMIDS software with HIV Predictive output and recommendation. This is the output of the Hausa-version of the WAHMIDS software showing prediction, prescription, recommendation and advice. This is S7 Figure_ Fig legend. S8 Figure. Igbo Language GUI of WAHMIDS software with sample HIV symptoms selection. This pink-colored Graphical User Interface (GUI) repr [file pone.0332573.s015.docx]

Appendix 2 – **All Supplementary Figures used in the manuscript.**


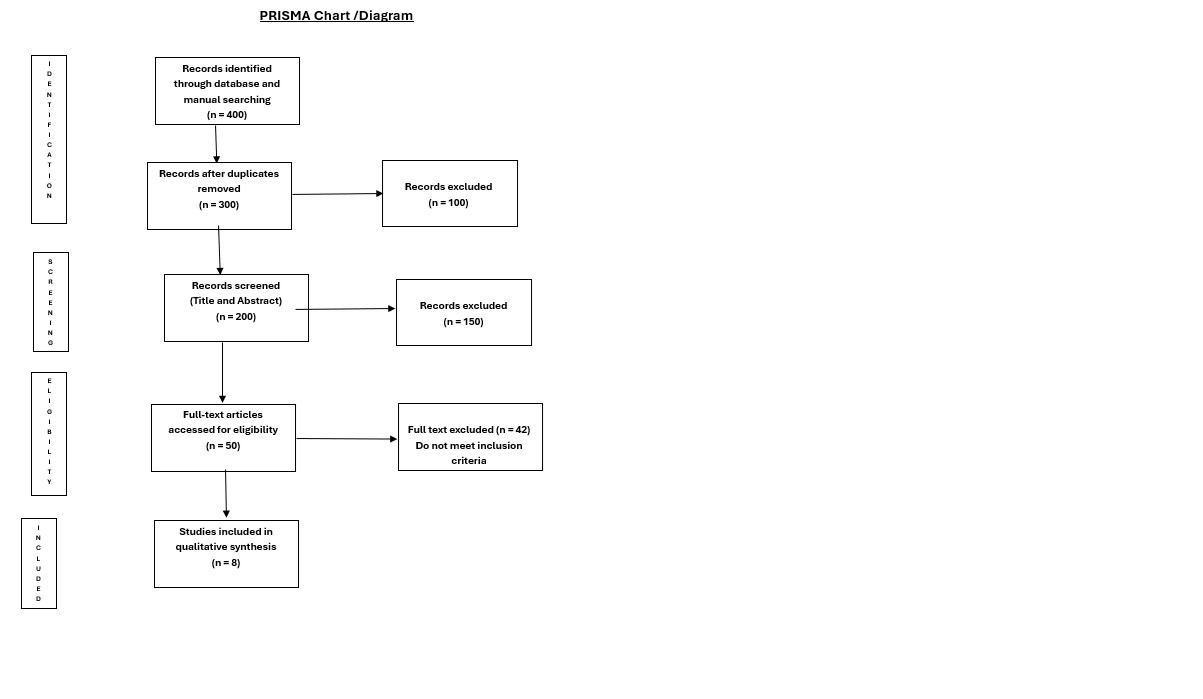


**S1 Figure: PRISMA flow diagram of the identification process for the sample of 8 articles**. The figure shows the systematic process of identifying the synthesized articles. This is S1 Figure_ Fig legend.


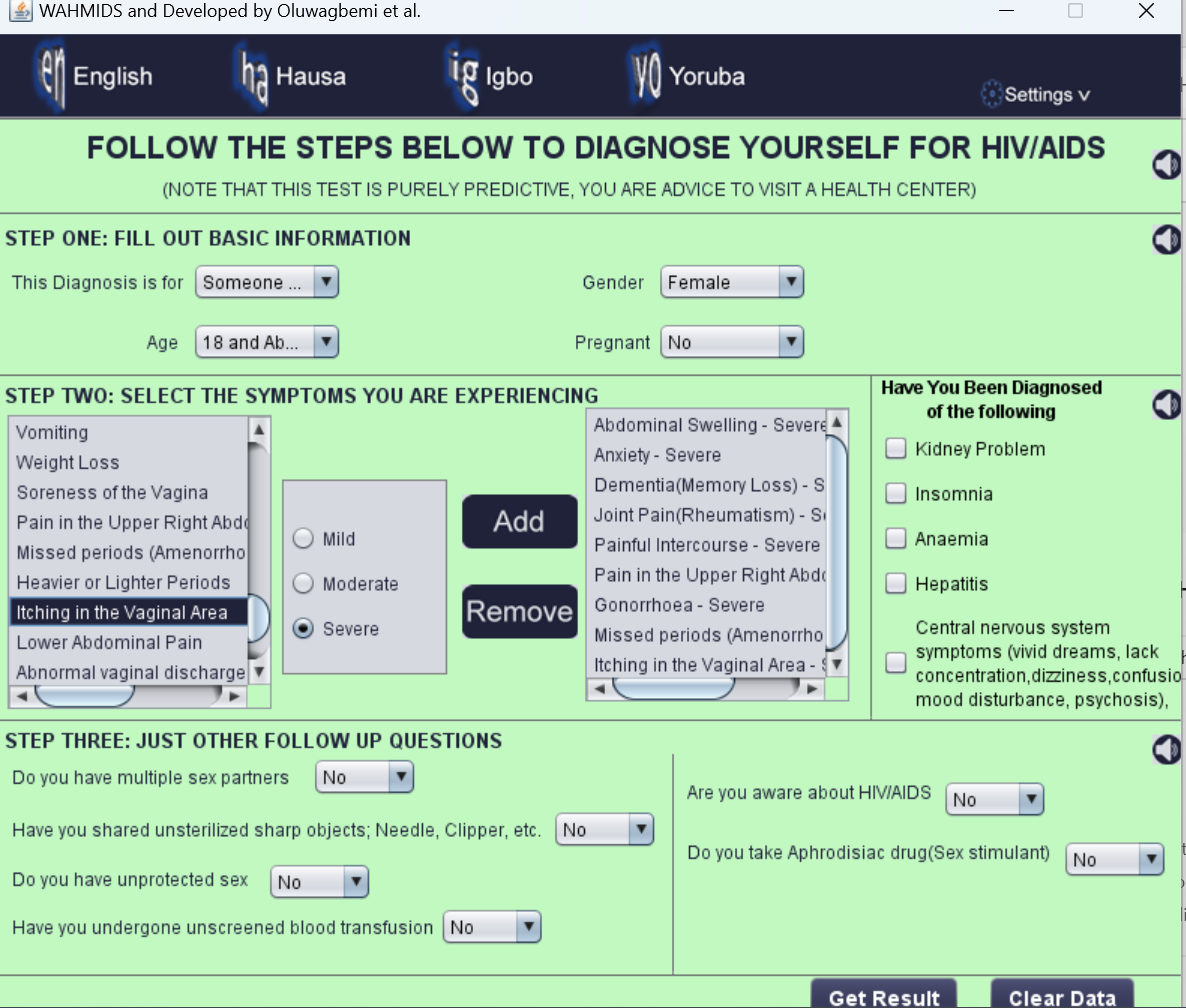


**S2 Figure. ENGLISH GUI of WAHMIDS software with sample HIV symptoms selection.**


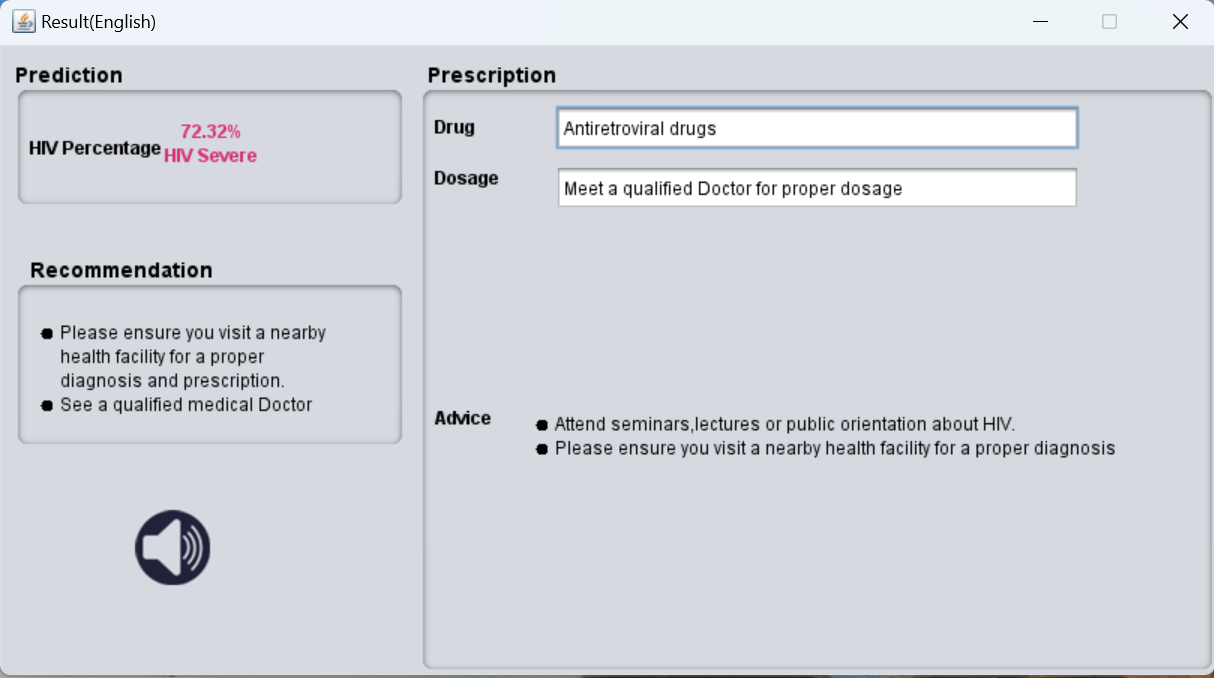


**S3 Figure. English GUI of WAHMIDS software with HIV Predictive output and recommendation.**


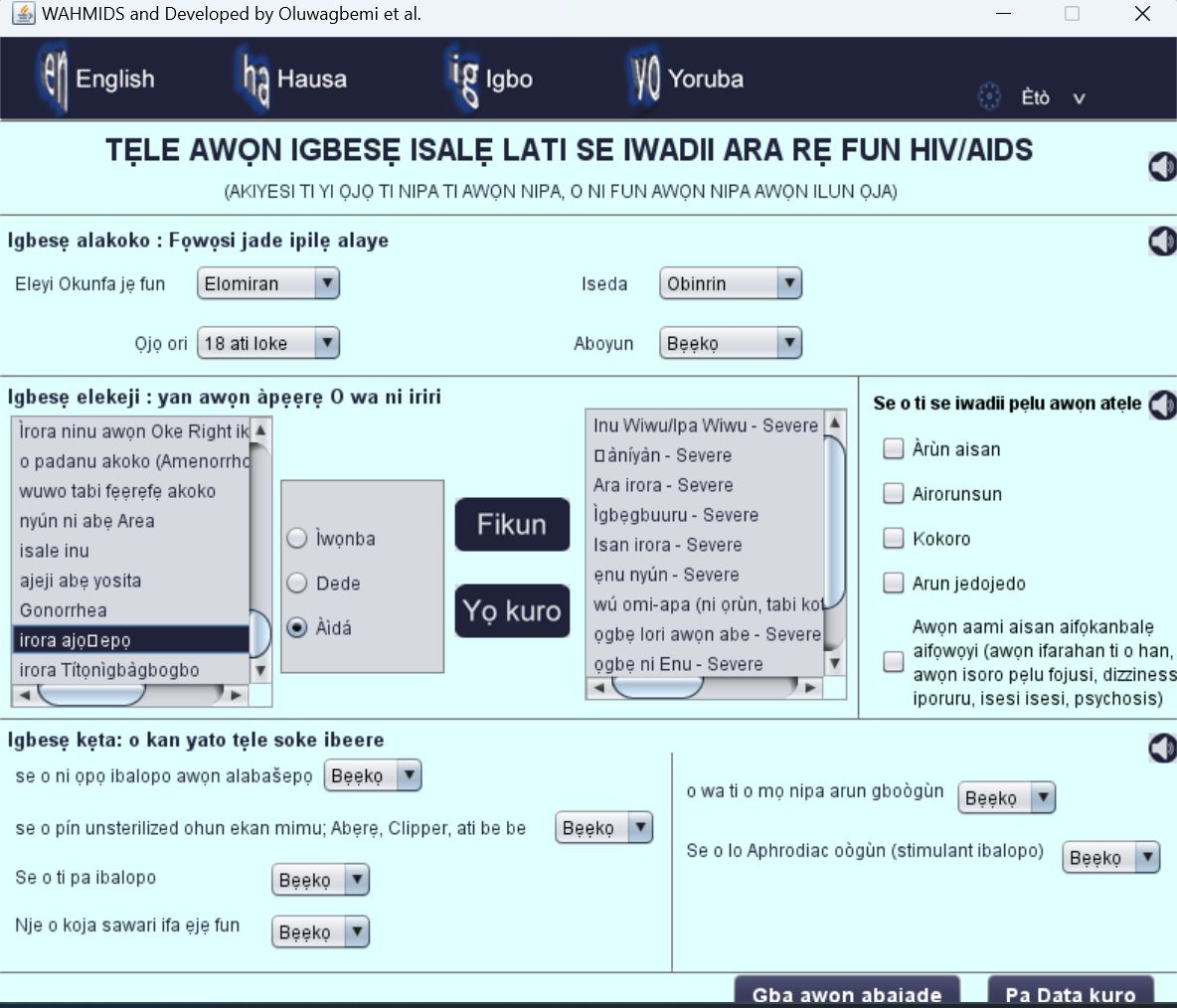


**S4 Figure. YORUBA GUI of WAHMIDS software with sample HIV symptoms selection.**


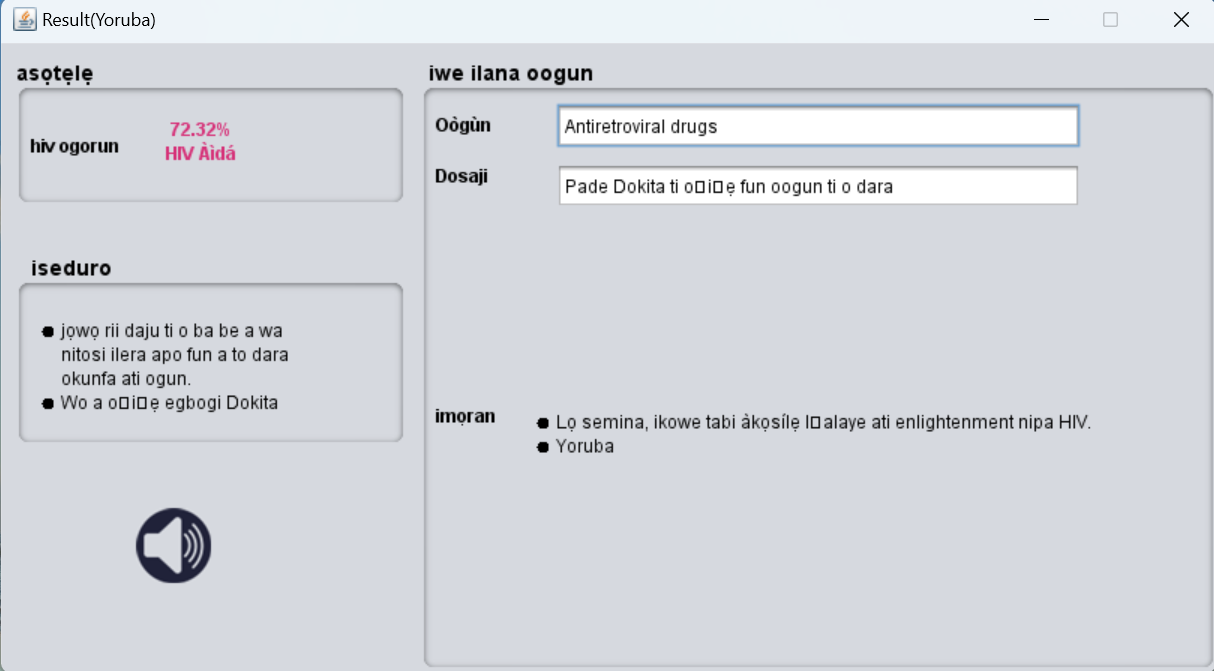


**S5 Figure**. **YORUBA GUI of WAHMIDS software with HIV Predictive output and recommendation.**


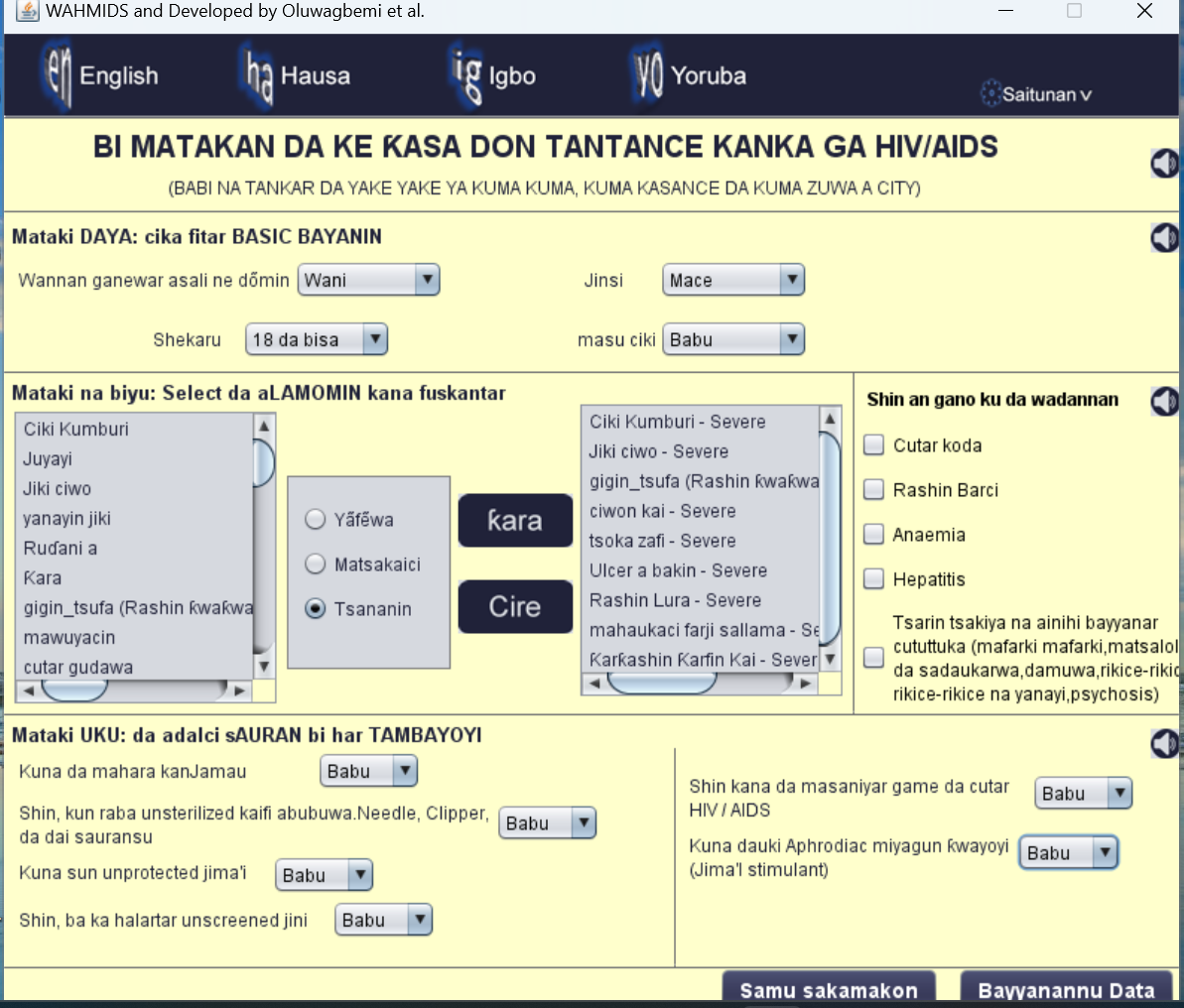


**S6 Figure. Hausa GUI of WAHMIDS software with sample HIV symptoms selection.**


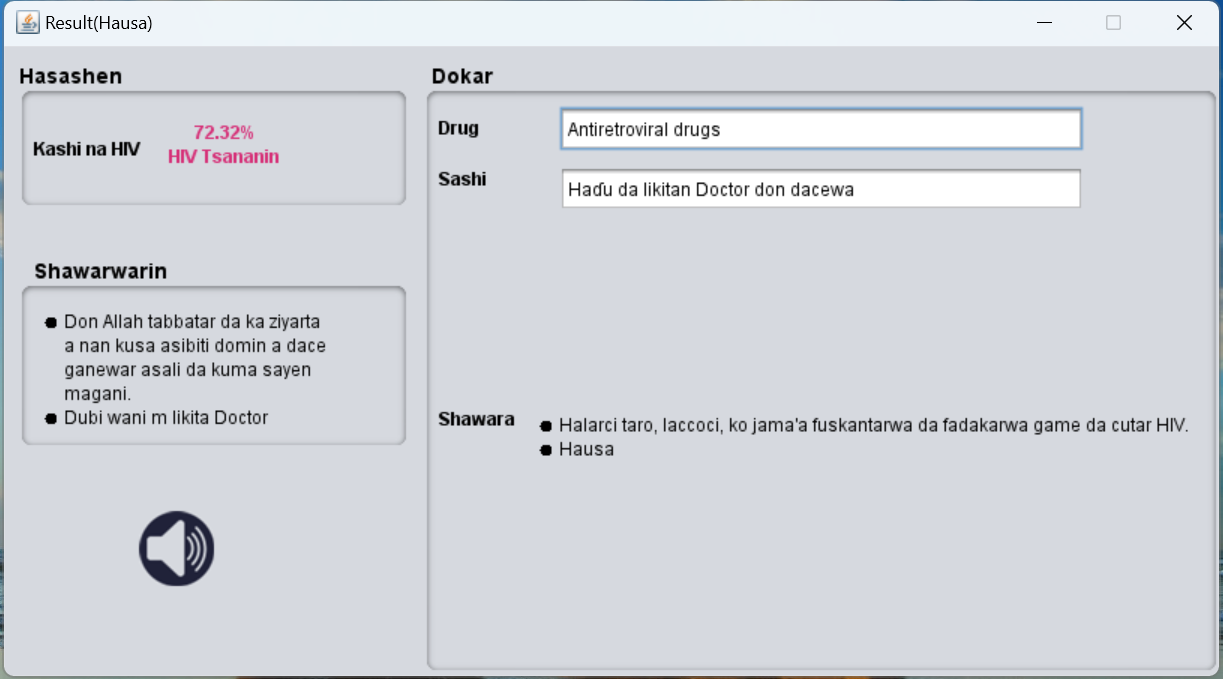


**S7 Figure. Hausa GUI of WAHMIDS software with HIV Predictive output and recommendation.**


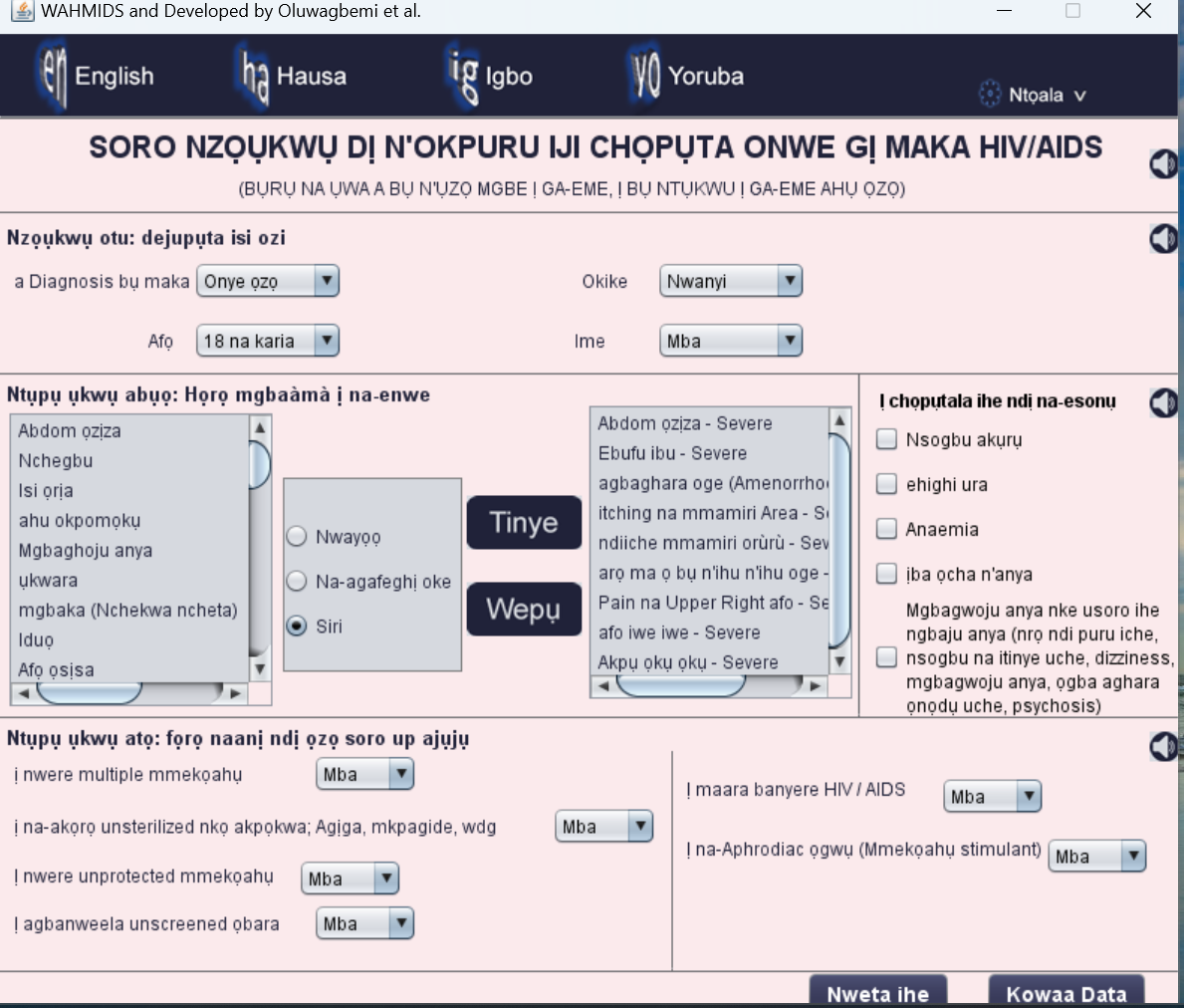


**S8 Figure. Igbo Language GUI of WAHMIDS software with sample HIV symptoms selection.**

**
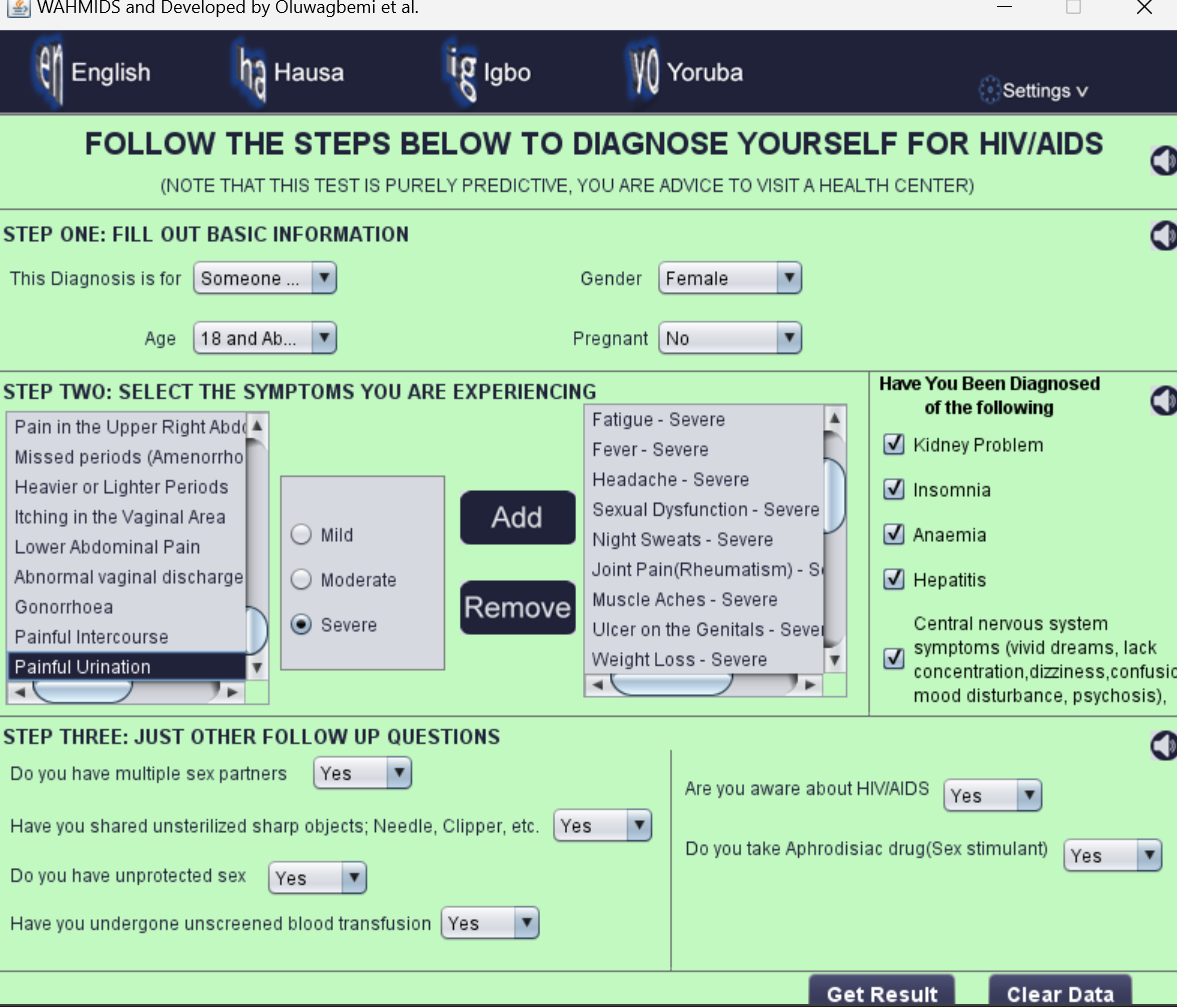
**

**S9 Figure. ENGLISH GUI of WAHMIDS software with sample HIV and HIV-comorbidity real-conditions selections.**


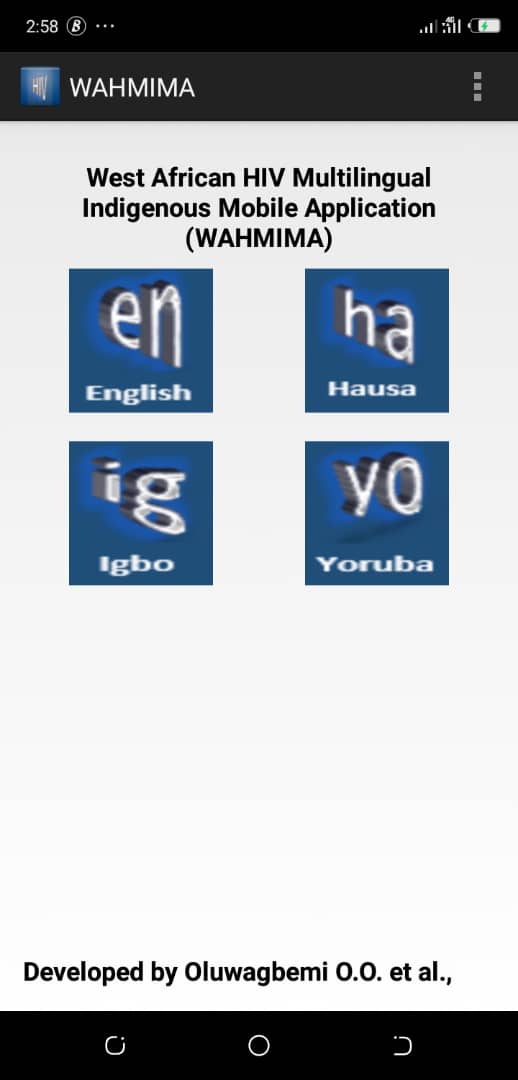


**S10 Figure. West African HIV Multilingual Indigenous Mobile Application (WAHMIMA) – Welcome page.**


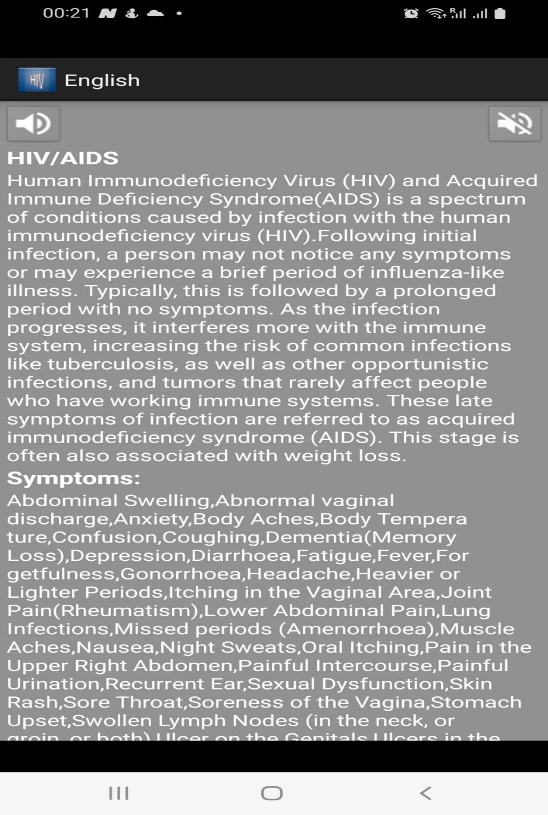


**S11 Figure. West African HIV Multilingual Indigenous Mobile Application (WAHMIMA) English Page 1.**


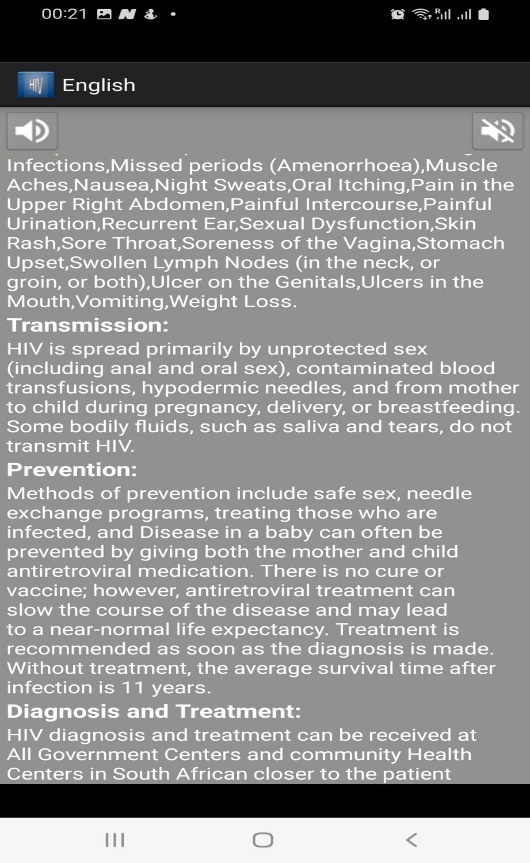


**S12 Figure. West African HIV Multilingual Indigenous Mobile Application (WAHMIMA) English Page 2.**


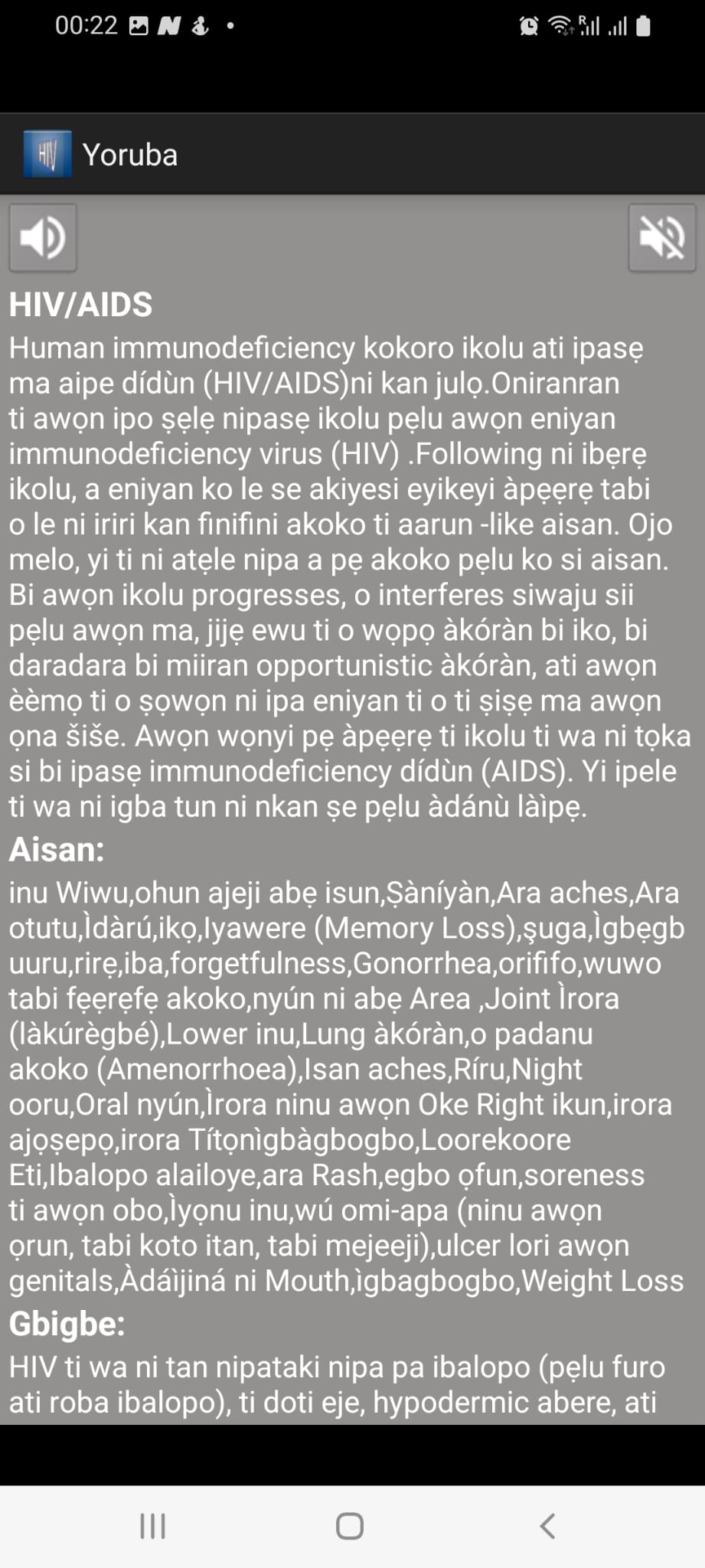


**S13 Figure. West African HIV Multilingual Indigenous Mobile Application (WAHMIMA) Yoruba Page 1.**


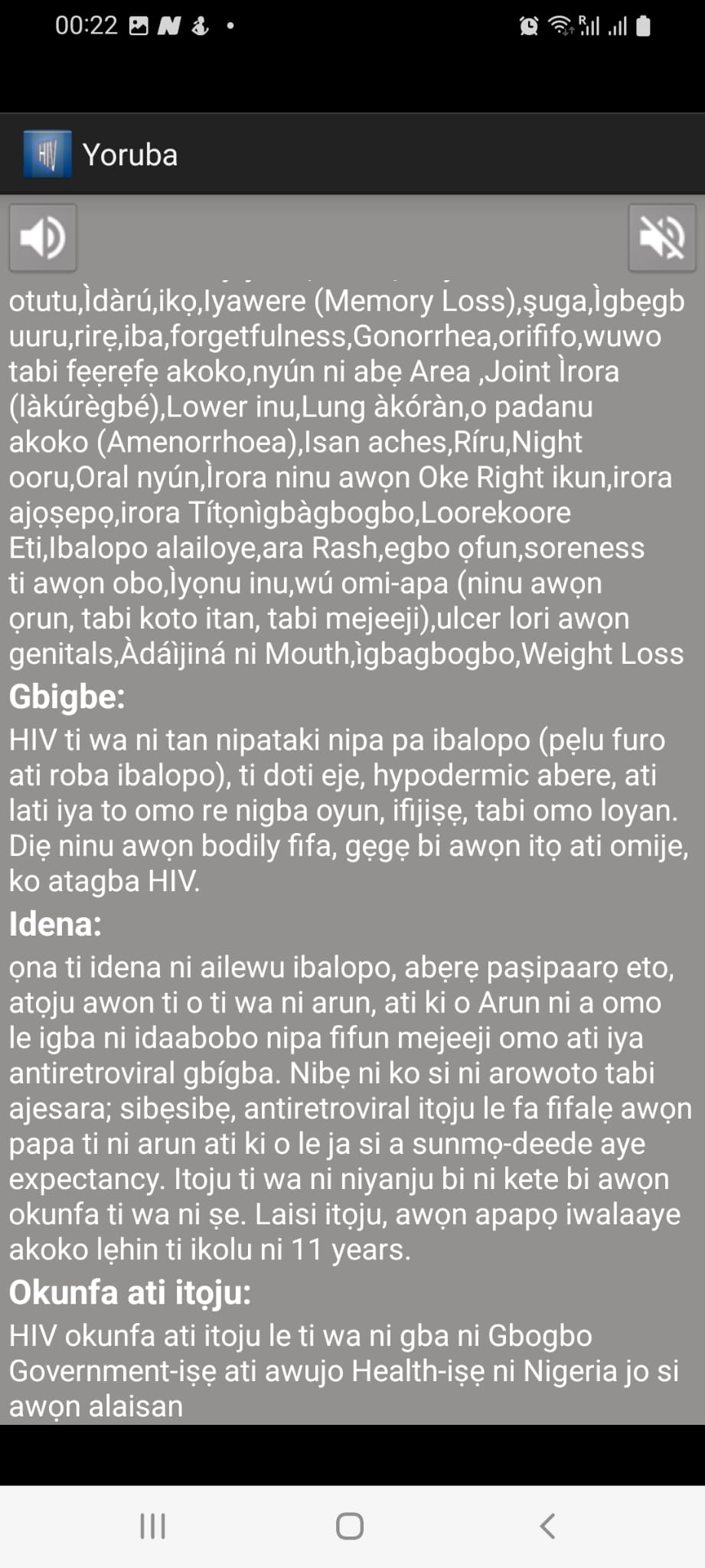


**S14 Figure. West African HIV Multilingual Indigenous Mobile Application (WAHMIMA) Yoruba Page 2.**


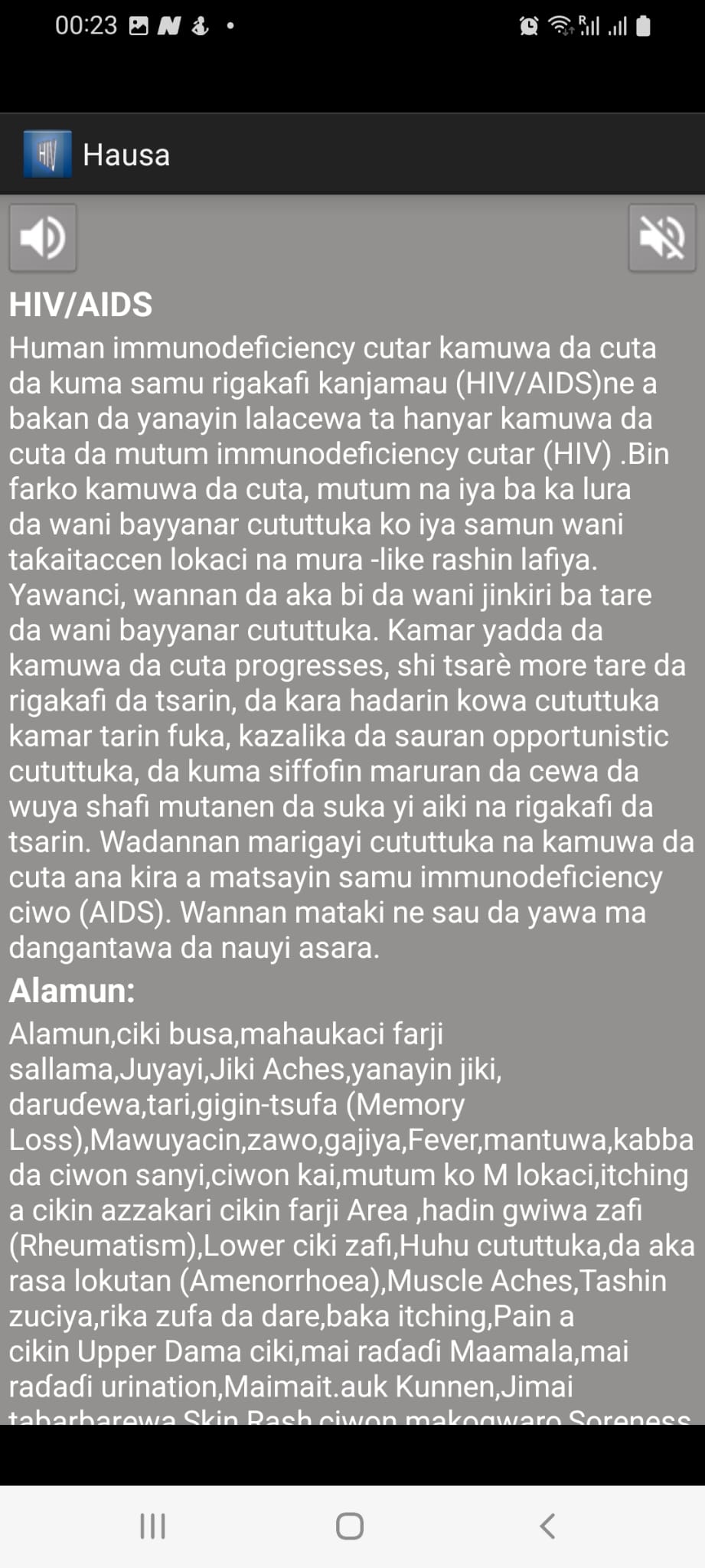


**S15 Figure. West African HIV Multilingual Indigenous Mobile Application (WAHMIMA) Hausa Page 1.**


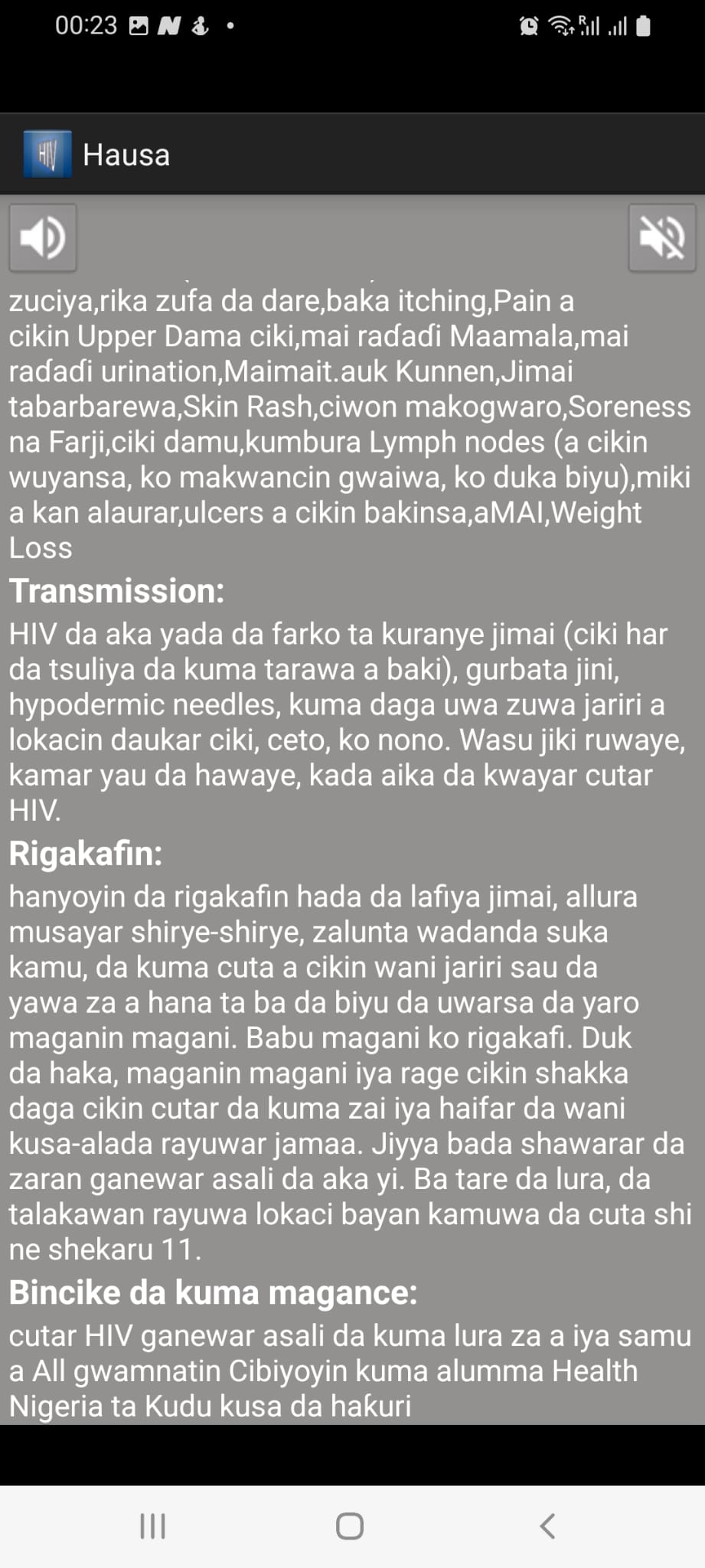


**S16 Figure. West African HIV Multilingual Indigenous Mobile Application (WAHMIMA) Hausa Page 2.**


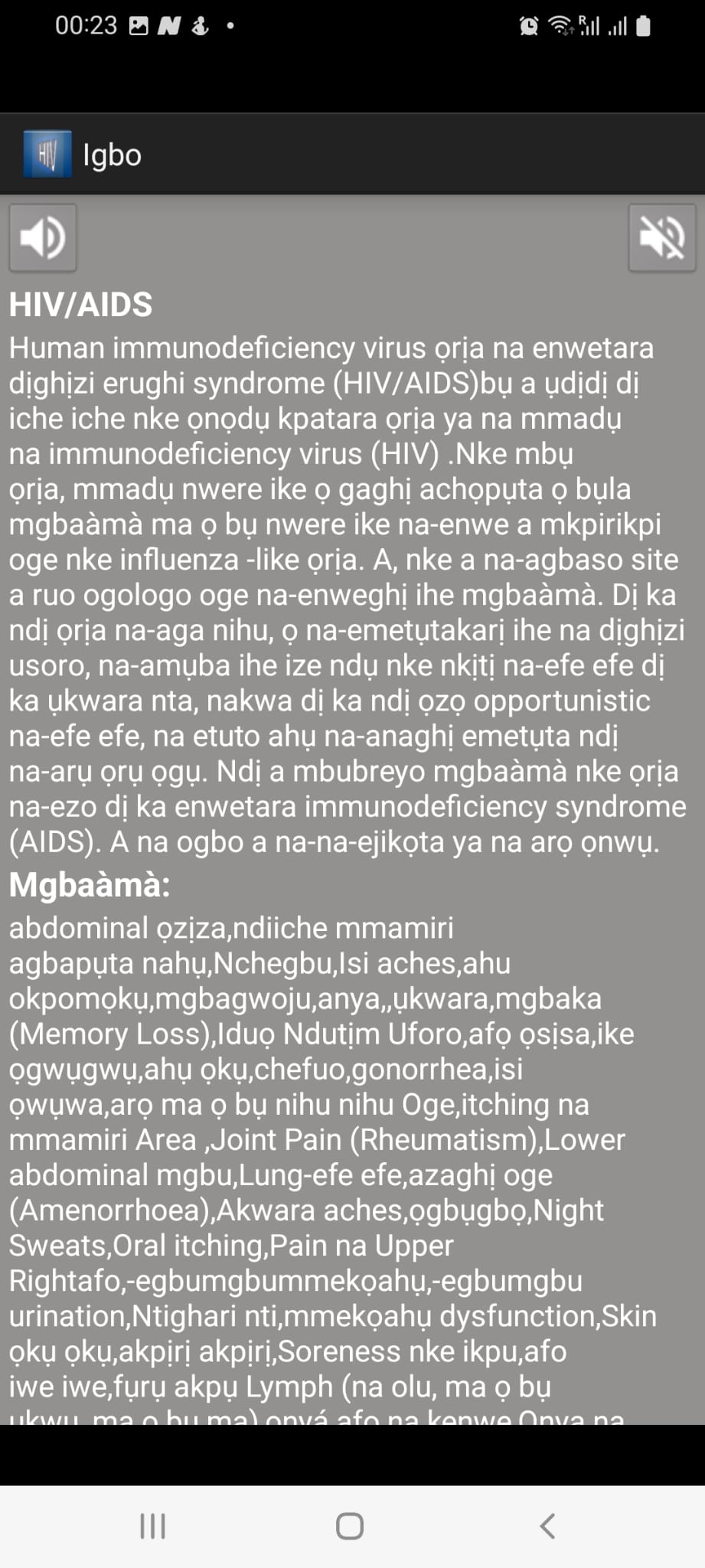


**S17 Figure. West African HIV Multilingual Indigenous Mobile Application (WAHMIMA) Igbo Page 1.**


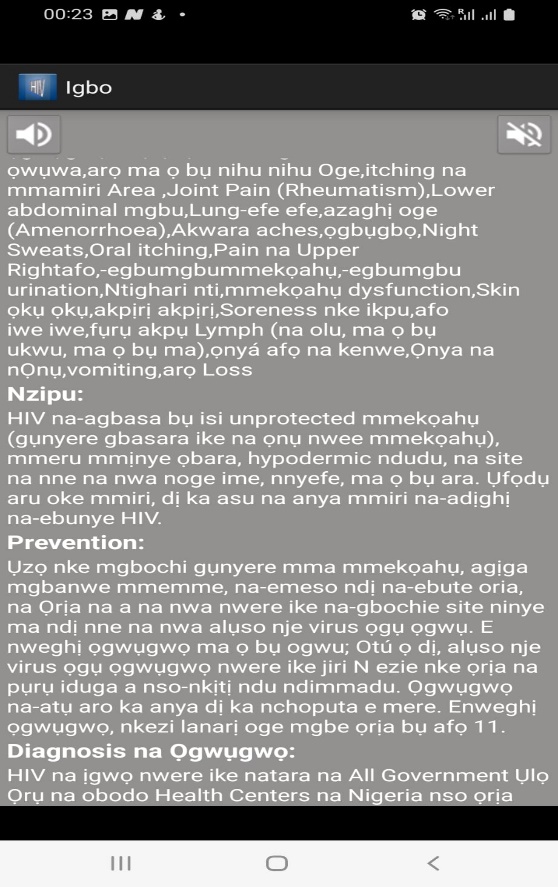


**S18 Figure. West African HIV Multilingual Indigenous Mobile Application (WAHMIMA) Igbo Page 2.**
